# p38α blocks brown adipose tissue thermogenesis through p38δ inhibition

**DOI:** 10.1371/journal.pbio.2004455

**Published:** 2018-07-06

**Authors:** Nuria Matesanz, Ivana Nikolic, Magdalena Leiva, Marta Pulgarín-Alfaro, Ayelén M. Santamans, Edgar Bernardo, Alfonso Mora, Leticia Herrera-Melle, Elena Rodríguez, Daniel Beiroa, Ainoa Caballero, Elena Martín-García, Rebeca Acín-Pérez, Lourdes Hernández-Cosido, Luis Leiva-Vega, Jorge L. Torres, Francisco Centeno, Angel R. Nebreda, José Antonio Enríquez, Rubén Nogueiras, Miguel Marcos, Guadalupe Sabio

**Affiliations:** 1 Fundación Centro Nacional de Investigaciones Cardiovasculares Carlos III, Madrid, Spain; 2 Department of Physiology, CIMUS, University of Santiago de Compostela-Instituto de Investigación Sanitaria, Santiago de Compostela, Spain; 3 CIBER Fisiopatología de la Obesidad y Nutrición (CIBERobn), Santiago de Compostela, Spain; 4 Bariatric Surgery Unit, Department of General Surgery, University Hospital of Salamanca, Salamanca, Spain; 5 Department of Internal Medicine, University Hospital of Salamanca-IBSAL, Department of Medicine, University of Salamanca, Salamanca, Spain; 6 Facultad de Ciencias, University of Extremadura, Grupo GIEN (Grupo de Investigación en Enfermedades Neurodegenerativas), Badajoz, Spain; 7 Institute for Research in Biomedicine (IRB Barcelona), The Barcelona Institute of Science and Technology, Barcelona, Spain; 8 ICREA, Barcelona, Spain; 9 CIBER Fragilidad y Envejecimiento Saludable (CIBERFES), Madrid, Spain; Harvard School of Public Health, United States of America

## Abstract

Adipose tissue has emerged as an important regulator of whole-body metabolism, and its capacity to dissipate energy in the form of heat has acquired a special relevance in recent years as potential treatment for obesity. In this context, the p38MAPK pathway has arisen as a key player in the thermogenic program because it is required for the activation of brown adipose tissue (BAT) thermogenesis and participates also in the transformation of white adipose tissue (WAT) into BAT-like depot called beige/brite tissue. Here, using mice that are deficient in p38α specifically in adipose tissue (p38α^Fab-KO^), we unexpectedly found that lack of p38α protected against high-fat diet (HFD)-induced obesity. We also showed that p38α^Fab-KO^ mice presented higher energy expenditure due to increased BAT thermogenesis. Mechanistically, we found that lack of p38α resulted in the activation of the related protein kinase family member p38δ. Our results showed that p38δ is activated in BAT by cold exposure, and lack of this kinase specifically in adipose tissue (p38δ ^Fab-KO^) resulted in overweight together with reduced energy expenditure and lower body and skin surface temperature in the BAT region. These observations indicate that p38α probably blocks BAT thermogenesis through p38δ inhibition. Consistent with the results obtained in animals, p38α was reduced in visceral and subcutaneous adipose tissue of subjects with obesity and was inversely correlated with body mass index (BMI). Altogether, we have elucidated a mechanism implicated in physiological BAT activation that has potential clinical implications for the treatment of obesity and related diseases such as diabetes.

## Author summary

Accumulation of fat in adipose tissue is essential to store energy and insulate the body; however, excessive body fat leads to obesity. Of the 2 existing types of adipose tissue, white adipose tissue (WAT) stores energy, whereas brown adipose tissue (BAT) can produce heat. Activation of BAT and transformation of WAT into brown-like ‘brite/beige’ adipocytes have recently emerged as novel strategies against obesity. The uncoupling protein 1 (UCP1) is a hallmark of BAT and is responsible for triggering these 2 processes under the regulation of the p38 MAP kinase (p38MAPK) pathway, but the underlying mechanisms remain unknown. Here, we have analysed this process in detail and demonstrate that a protein kinase called p38α directly correlates with UCP1 levels in human adipose tissue, while it inversely correlates with body mass index (BMI). We find that mice lacking p38α in adipose tissue are protected against diet-induced obesity due to increased body temperature. In addition, another p38 family member, p38δ, is activated in these adipocytes lacking p38α and reduces their thermogenic capacity. Our results suggest that these 2 members of the p38 family have opposite roles in controlling thermogenesis.

Obesity is a serious worldwide health problem, associated with a higher risk of life-threatening diseases [2], that has had a dramatic increase in prevalence [1]. As the main organ for fat storage, adipose tissue has a fundamental role in metabolism [3]. Whereas white adipose tissue (WAT) stores energy in the form of triglycerides and releases free fatty acids on demand, brown adipose tissue (BAT) burns fat to maintain the temperature in a process called non-shivering thermogenesis [4]. Classically, it was assumed that in adult humans BAT played a minor role in energy metabolism. However, recent findings have indicated that this tissue can be modulated by several stimuli presenting lower activity in individuals with obesity [5–7]. Additionally, under certain stimuli, WAT can increase its thermogenic capacity in a process called browning [8–11]. This remodelling of WAT has acquired special interest because it has important therapeutic implications in the treatment of obesity [12, 13].

The p38MAPK pathway is activated during browning, and it has been suggested that this drives adipose tissue remodelling [14, 15]. There are 4 p38 isoforms: p38α, p38β, p38γ, and p38δ, all of which are activated by stress stimuli in a cell-dependent manner, controlling cellular fate [16–20]. It has been extensively described that p38MAPK triggers browning and BAT activation through the transcription of uncoupling protein 1 (UCP1) via cAMP response element-binding (CREB), activating transcription factor 2 (ATF2), and peroxisome proliferator-activated receptor gamma coactivator 1α (PGC1α) activation. In fact, β-adrenergic stimulation and other browning agents stimulate the p38MAPK cascade, promoting thermogenesis [18, 21–23]. Although most of these studies assumed that the phenotype is driven by p38α, the specific role of the isoform p38α and other p38 isoforms in the development and transformation of adipose tissue has not been elucidated yet using genetically modified mouse models.

Using conditional animals for p38α (p38α^Fab-KO^), unexpectedly, we found that deletion of this kinase in adipose tissue protected animals against high-fat diet (HFD)-induced obesity together with increased energy expenditure followed by higher BAT thermogenesis. Lack of p38α in BAT resulted in higher activation of p38δ. In agreement with this, conditional deletion of p38δ in adipose tissue led to obesity, with higher body weight and reduced energy expenditure due to a lower body and skin surface temperature in the BAT region. Besides, lack of p38α in inguinal fat (iWAT) increased p38γ activation and UCP1 expression. Our results indicate that p38α controls p38δ activation in BAT, regulating thermogenesis and energy expenditure. In contrast, in WAT, p38α would have opposite effects depending on the fat depot, blocking browning through inhibition of p38γ in iWAT and promoting browning in epididymal fat (eWAT). Thus, these findings challenge the classical view of p38α as an activator of BAT thermogenesis. These studies provided important insights into p38δ and p38α function in BAT regulation that could have therapeutic implications to efficiently fight obesity.

## Results

**Fig 1:**
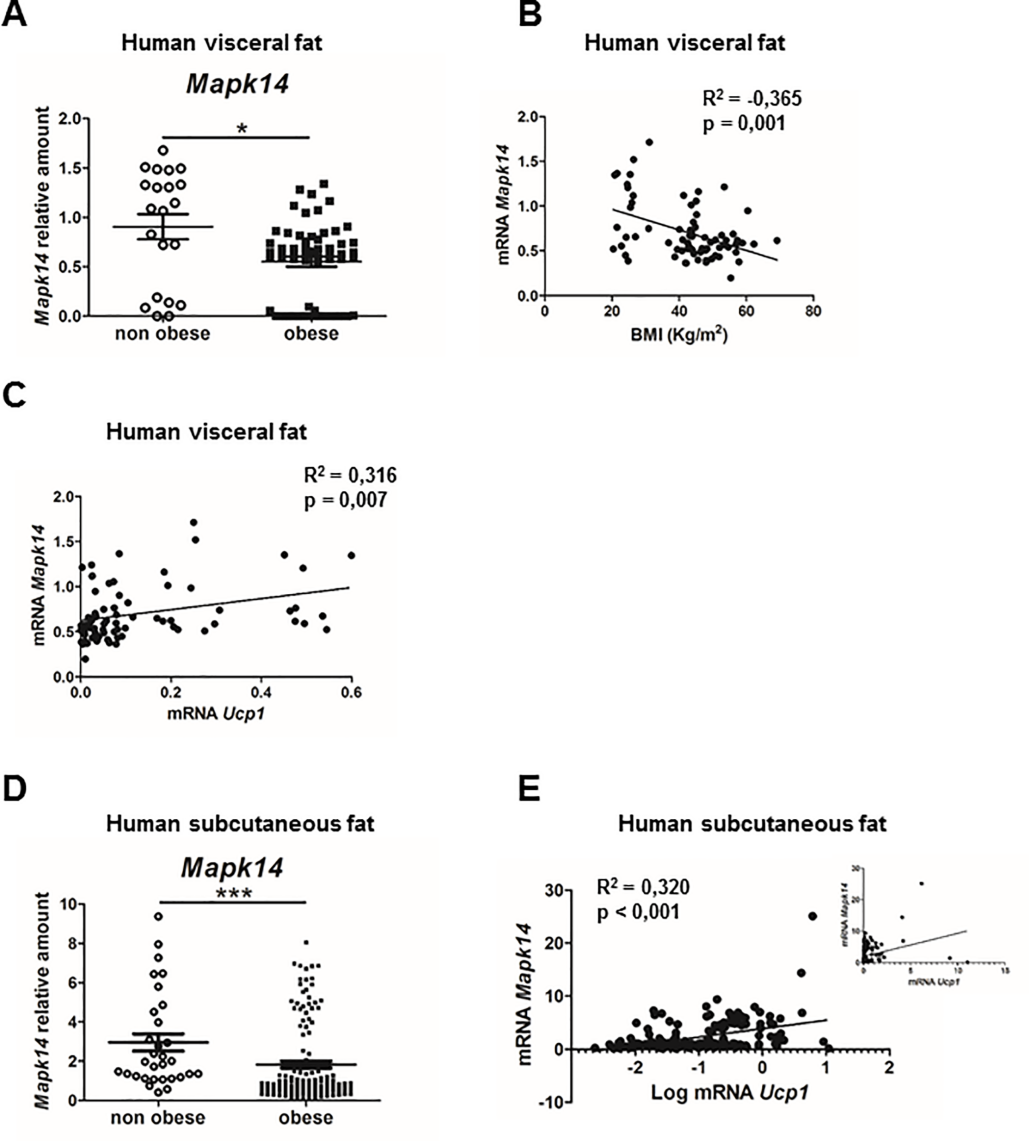
p38α in human visceral fat inversely correlated with BMI and directly correlated with UCP1 in human visceral fat and sWAT. (A) mRNA levels of *Mapk14* (p38α) in visceral fat from lean individuals and individuals with obesity—mRNA expression was normalised to the amount of *Gapdh* mRNA. (B) Correlation between mRNA levels of *Mapk14* (p38α) and BMI (r^2^ = −0,365; *p* = 0.001) or (C) *Ucp1* in visceral fat (r^2^ = 0.316; *p* = 0.007). The mRNA levels of *Mapk14* (p38α) and *Ucp1* were determined by qRT-PCR (*n* = 71). (D) mRNA levels of *Mapk14* (p38α) in sWAT from lean individuals and individuals with obesity. mRNA expression was normalised to the amount of *Gapdh* mRNA. (E) Correlation between mRNA levels of *Mapk14* (p38α) and *Ucp1* in sWAT (r^2^ = 0.320; *p* < 0.0001). Graph correlating mRNA *Mapk14* and log mRNA *Ucp1* is also shown. The mRNA levels of *Mapk14* (p38α) and *Ucp1* were determined by qRT-PCR (*n* = 168). See also S1 Data. Linear relationships between variables were tested using Pearson’s correlation coefficient. BMI, body mass index; qRT-PCR, quantitative real-time polymerase chain reaction; UCP1, uncoupling protein 1.

p38α has emerged as one of the main player that could activate the thermogenic capacity of adipose tissue. Because the thermogenesis of adipose tissue is reduced in obesity [6, 7, 21], we wondered whether expression of this kinase changes in human WAT during obesity. Using 2 cohorts for visceral fat and subcutaneous fat (sWAT) of adult patients with 80 and 170 samples, respectively, we found that the expression of p38α (*Mapk14*) in visceral fat and sWAT from individuals with obesity was reduced compared with those without obesity (Fig 1A and 1D). In fact, mRNA levels of *Mapk14* in visceral fat inversely correlated with body mass index (BMI) (Fig 1B). It has been suggested that p38α in WAT activates browning by triggering the expression of UCP1 [18], the main protein responsible for adipose tissue thermogenic capacity [22]. In visceral fat and sWAT from individuals with obesity and those without obesity, we found that expression of *Mapk14* correlated positively with the levels of *Ucp1* (Fig 1C and 1E). This correlation reinforced the idea that p38α in visceral fat and sWAT controls the levels of UCP1 and could regulate browning in humans.

Then, we evaluated the function of p38α in adipose tissue using conditional mice (p38α^Fab-KO^), which lacked p38α in WAT and BAT (S1 Fig). Under normal-chow diet (ND), p38α^Fab-KO^ mice had the same weight gain as the control Fab-Cre mice (S2A Fig). However, they presented reduced fat mass, in concordance with lower eWAT, perirenal WAT (pWAT), and BAT weight (S2B and S2C Fig). This reduction in fat accumulation was associated with higher energy expenditure and slight increase of body temperature (S2G and S2H Fig). In fact, these mice presented lower blood glucose levels in fasted and fed conditions (S2D Fig) and increased glucose tolerance (S2E Fig), with no differences in insulin sensitivity or insulin-stimulated glucose transporter type 4 (GLUT4) translocation in adipose tissue (S2E and S2F Fig). These data suggest that lack of p38α might protect against type 2 diabetes. Moreover, we evaluated whether lack of p38α affects adipogenesis, browning, and metabolism in eWAT and BAT. BAT from p38α^Fab-KO^ mice presented an increase of *Cidea*, a marker of browning, together with higher expression of glycolytic and β oxidation genes (S3 Fig).

**Fig 2:**
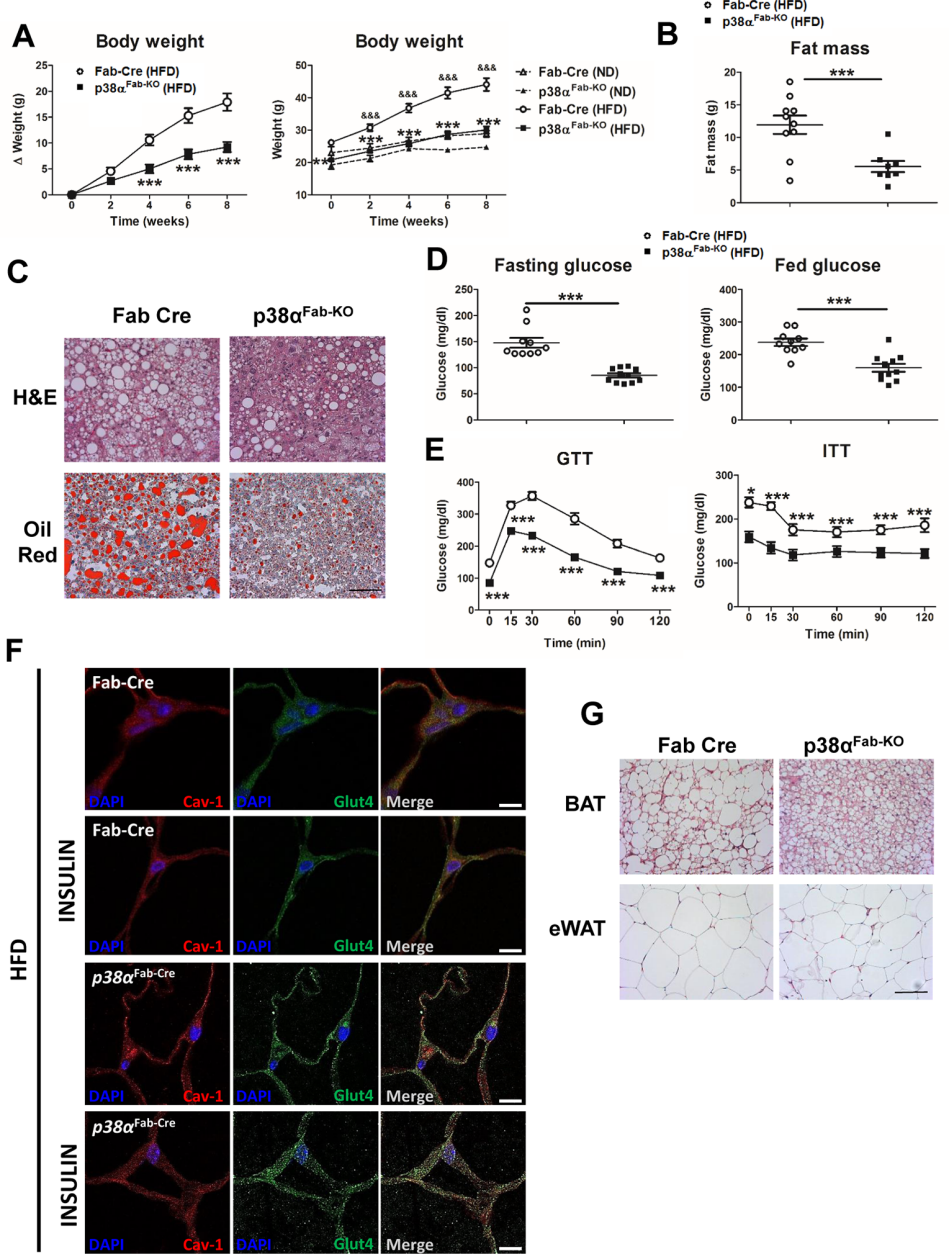
p38α^Fab-KO^ mice are protected against diet-induced obesity and diabetes. (A) Body weight time course in Fab-Cre and p38α^Fab-KO^ male (8–10-wk-old) mice fed an HFD over 8 weeks. Data are presented as the increase above initial weight (left panel) or as total weight comparing mice fed an HDF with mice fed an ND (right panel). HFD-induced weight gain was significantly higher in Fab-Cre than p38α^Fab-KO^ mice (mean ± SEM; Fab-Cre HFD *n =* 10 mice; p38α^Fab-KO^ HFD *n =* 11 mice; Fab-Cre ND *n =* 9 mice; p38α^Fab-KO^ ND *n =* 8 mice). (B) NMR analysis of fat mass in p38α^Fab-KO^ and Fab-Cre mice after 8 weeks of HFD (mean ± SEM; Fab-Cre *n =* 10 mice; p38α^Fab-KO^ *n =* 8 mice). (C) Representative haematoxylin–eosin and oil red O staining of liver sections (Fab-Cre *n =* 6 mice; p38α^Fab-KO^ *n =* 6 mice; and 3 pictures from each mouse). Scale bar: 50 μm. (D) Fasting and fed blood glucose in Fab-Cre and p38α^Fab-KO^ mice fed the HFD (8 weeks) (mean ± SEM; Fab-Cre *n =* 10 mice; p38α^Fab-KO^ *n =* 11 mice). (E) GTT and ITT in Fab-Cre and p38α^Fab-KO^ mice fed the HFD for 8 weeks. Mice were fasted overnight (for GTT) or 1 hour (for ITT), and blood glucose concentration was measured in mice given intraperitoneal injections of glucose (1 g/kg of total body weight) or insulin (0.75 U/kg of total body weight) (mean ± SEM; Fab-Cre *n =* 10 mice; p38α^Fab-KO^ *n =* 11 mice). (F) Immunohistochemistry of eWAT sections using anti-GLUT4 (green), anti-Cav-1 (red) antibodies, and the nuclear dye DAPI (blue). Location of GLUT4 was analysed in mice treated without or with insulin (1.5 IU/kg) for 15 minutes after overnight fasting. Scale bar: 20 μm. (G) Representative haematoxylin–eosin BAT and eWAT sections (Fab-Cre *n =* 6 mice; p38α^Fab-KO^ *n =* 6 mice; and 3 pictures from each mouse). Scale bar: 50 μm. *p < 0.05, ***p < 0.001 Fab-Cre versus p38α^Fab-KO^. ‘&&’ indicates *p* < 0.01, ‘&&&’ indicates *p* < 0.001 Fab-Cre ND versus Fab-Cre HFD (2-way ANOVA coupled with Bonferroni’s post-tests or *t* test or Welch’s test when variances were different). See also S1 Data. BAT, brown adipose tissue; Cav-1, caveolin-1; eWAT, epididymal fat; GLUT4, glucose transporter type 4; GTT, glucose tolerance test; HFD, high-fat diet; ITT, insulin tolerance test; ND, normal-chow diet; WAT, white adipose tissue.

To further evaluate the role of p38α in adipose tissue, mice were fed an HFD, and we observed that p38α^Fab-KO^ mice were completely protected from diet-induced obesity because their weight was identical to the weight of the control animals in ND (Fig 2A). This reduced weight gain was in line with lower fat mass (Fig 2B) and reduced weight of the different fat depots, including eWAT, sWAT, iWAT, pWAT, and BAT (S4A Fig). Moreover, liver weight was also reduced in agreement with protection against HFD-induced liver steatosis in p38α^Fab-KO^ mice (Fig 2C and S4A Fig). The protection against HFD-induced obesity was associated with reduced fasted and fed hyperglycaemia in p38α^Fab-KO^ mice, with no differences in triglyceridemia (Fig 2D and S4E Fig). In addition, p38α^Fab-KO^ mice were protected against HFD-induced glucose intolerance even when glucose dose was adjusted to lean mass (Fig 2E, S4B Fig.) and insulin resistance as shown by the reduced glucose levels during the insulin tolerance test (ITT) (Fig 2E). HFD-induced obesity was associated with liver insulin resistance and reduced insulin-stimulated Akt phosphorylation in livers from HFD-fed Fab-Cre mice (S4C Fig). Evaluation of insulin sensitivity in several tissues indicated that HFD-fed p38α^Fab-KO^ mice presented higher insulin-induced phosphorylation of Akt at Thr308 and Ser473 than HFD-fed Fab-Cre mice in liver and muscle but not in eWAT nor BAT (S4D Fig). Furthermore, we observed a slight increase of insulin-stimulated GLUT4 translocation in eWAT (Fig 2F). Together, these results demonstrate that p38α^Fab-KO^ mice are protected against diet-induced obesity and diabetes.

**Fig 3:**
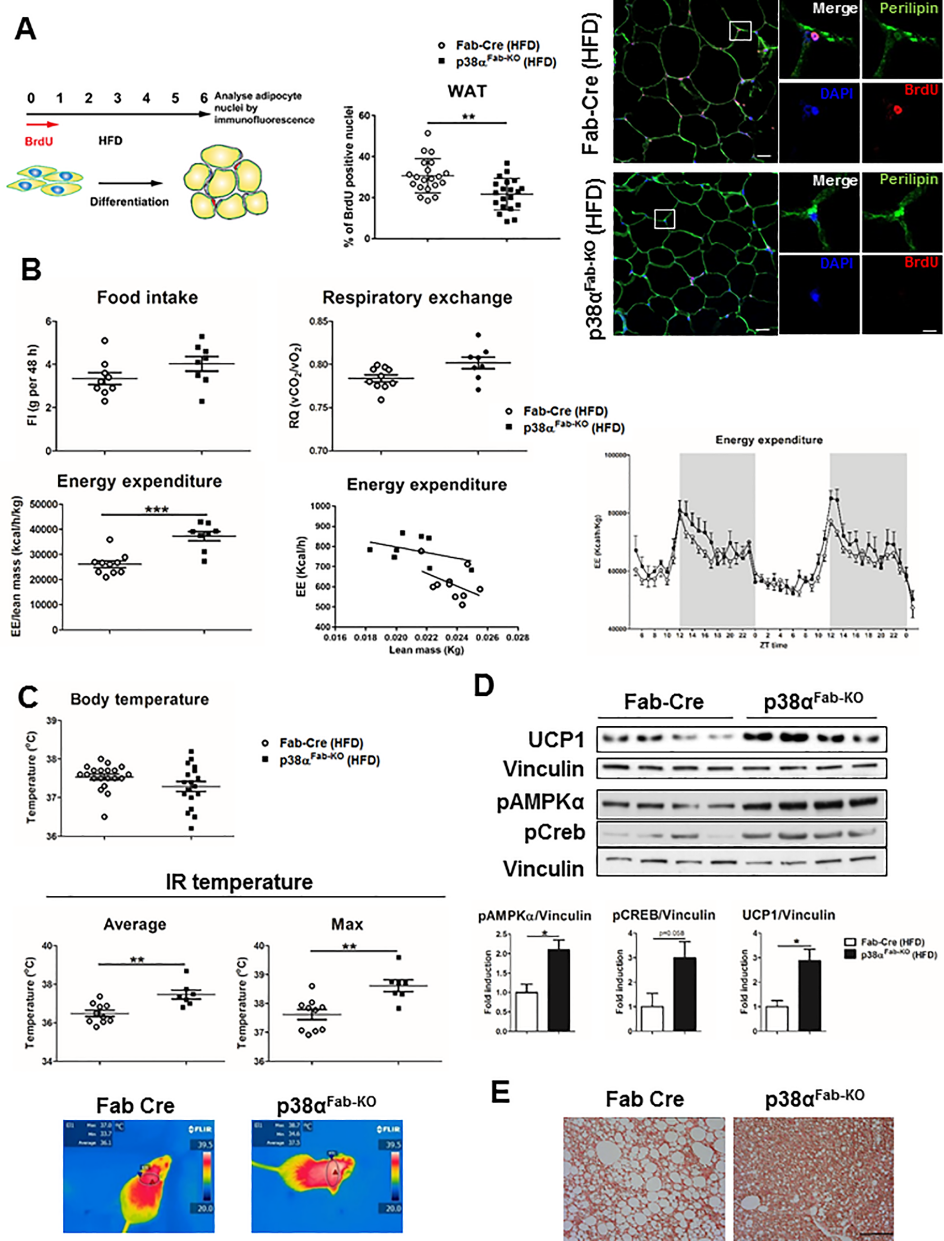
p38α^Fab-KO^ mice have higher energy expenditure and increased BAT thermogenesis. Fab-Cre and p38α^Fab-KO^ mice were fed an HFD for 8 weeks. (A) Analysis of eWAT expansion in HFD-fed Fab-Cre and p38α^Fab-KO^ mice. Animals were treated with BrdU in the drinking water during the first week of a 6-week HFD. Cartoon explaining the protocol is shown in the left panel. BrdU incorporation into the nuclei was detected by immunofluorescence in eWAT sections (right panel). Cell outlines were stained with anti-perilipin antibody (green) and nuclei, with DAPI (blue). Scale bar: 20 μm. A cell in detail is shown in a bigger magnification for each genotype. Quantification of positive BrdU nuclei is showed in the middle panel. (B) Comparison of energy balance between HFD-fed Fab-Cre and p38α^Fab-KO^ mice. HFD-fed mice were examined in a metabolic cage over a 2-day period to measure FI, respiratory exchange, and EE. FI and EE (left) over 2 days were corrected by lean mass. EE expressed as ANCOVA analysis (middle panel) and hour by hour over 48-h period (right panel) are also shown (mean ± SEM; Fab-Cre *n =* 10 mice; p38α^Fab-KO^ *n =* 8 mice). (C) Body (mean ± SEM; Fab-Cre *n =* 20 mice; p38α^Fab-KO^ *n =* 18 mice) and skin temperature of surrounding interscapular BAT (mean ± SEM; Fab-Cre *n =* 10 mice; p38α^Fab-KO^ *n =* 7 mice). Lower panels show representative infrared thermal images. (D) Immunoblot analysis of UCP1 levels and Creb and AMPK phosphorylation in lysates from BAT. Quantification is shown in the lower panel. (E) Immunohistochemistry staining of UCP1 after 8 weeks of HFD in BAT. Scale bar: 50 μm. Statistically significant differences between Fab-Cre and p38α^Fab-KO^ mice are indicated: **p < 0.01 (*t* test or Welch’s test when variances were different). See also S1 Data. AMPK, 5' adenosine monophosphate-activated protein kinase; BAT, brown adipose tissue; BrdU, bromodeoxyuridine; Creb, cAMP response element-binding; EE, energy expenditure; eWAT, epididymal fat; FI, food intake; HFD, high-fat diet; IR temperature, infrared temperature; UCP1, uncoupling protein 1; WAT, white adipose tissue.

Histological analysis showed that interscapular BAT depot from HFD-fed p38α^Fab-KO^ mice had small multilocular adipocytes (Fig 2G), whereas in eWAT, we observed a slight decrease of adipocyte size (Fig 2G), which correlates to reduced cell size in BAT and WAT adipocytes from HFD-fed p38α^Fab-KO^ with respect to HFD-fed Fab-Cre (S5A and S5C Fig). Then, we evaluated HFD-induced WAT adipocyte expansion by bromodeoxyuridine (BrdU) staining [23], observing reduced expansion in p38α^Fab-KO^ (Fig 3A). However, no differences in Ki67 staining were observed after HFD in WAT or BAT adipocytes (S5A and S5C Fig).

To further investigate the mechanism by which lack of p38α in adipose tissue could protect against HFD-induced obesity, we evaluated whole-body metabolism using metabolic cages. HFD-fed p38α^Fab-KO^ mice showed a significant increase in whole-body energy expenditure analysed by ANCOVA, with no changes in food intake or respiratory exchange ratio (Fig 3B). These data are consistent with the observation that HFD-fed p38α^Fab-KO^ mice have higher skin temperature in the region of BAT compared with Fab-Cre mice (Fig 3C). Western blot analysis of BAT indicated that HFD-fed p38α^Fab-KO^ mice presented a slight increase of UCP1 expression associated with higher AMPK and Creb phosphorylation (Fig 3D and 3E). In addition, higher expression of UCP1 levels was observed in iWAT from HFD-fed p38α^Fab-KO^ mice (S5B and S7A Figs), suggesting an increased browning of this adipose depot. In contrast with the up-regulated UCP1 levels in iWAT, analysis of eWAT by western blot and immunohistochemistry showed that HFD-fed p38α^Fab-KO^ mice have reduced UCP1 levels in this tissue (S6 and S7B Figs). These results are in agreement with the results found in human visceral fat (Fig 1C) suggesting that, in visceral fat, p38α directly correlates with UCP1.

**Fig 4:**
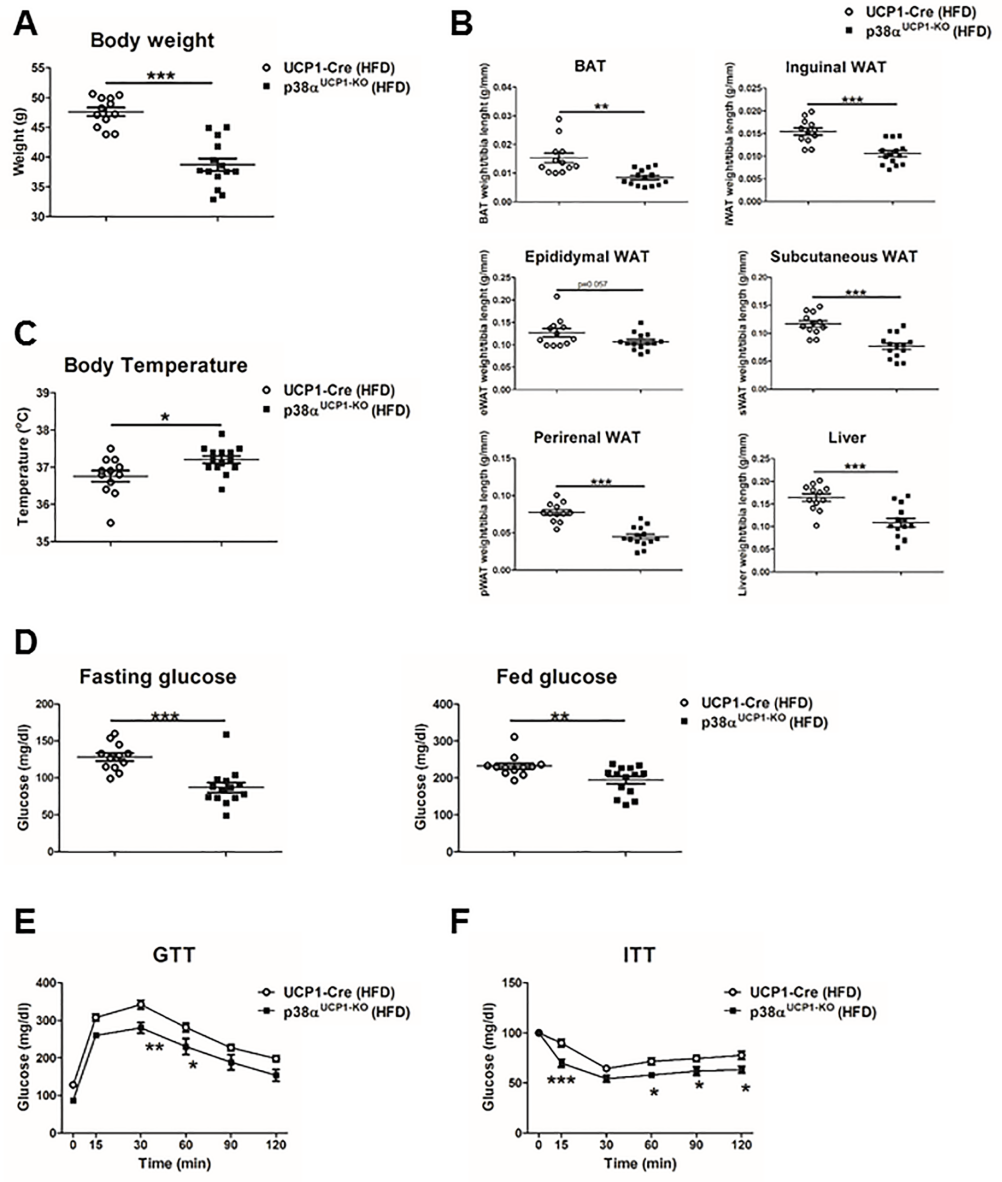
p38α controls BAT thermogenesis. UCP1-Cre and p38α^UCP1-KO^ mice were fed with HFD for 8 weeks. (A) Body weight at the end of the treatment (mean ± SEM; UCP1-Cre *n =* 12 mice; p38α^UCP1-KO^ *n =* 14 mice). (B) Weight of BAT, eWAT, sWAT, iWAT, pWAT, and liver relativised to tibia length (mean ± SEM; UCP1-Cre *n =* 12 mice; p38α^UCP1-KO^ *n =* 14 mice). (C) Body temperature of HFD-fed UCP1-Cre and p38α^UCP1-KO^ mice (mean ± SEM; UCP1-Cre *n =* 12 mice; p38α^UCP1-KO^ *n =* 14 mice). (D) Fasting and fed blood glucose in UCP1-Cre and p38α^UCP1-KO^ mice fed an HFD (8 weeks) (mean ± SEM; UCP1-Cre *n =* 12 mice; p38α^UCP1-KO^ *n =* 14 mice). (E) GTT and (F) ITT in UCP1-Cre and p38α^UCP1-KO^ mice fed HFD for 8 weeks. Mice were fasted overnight (for GTT) or 1 hour (for ITT), and blood glucose concentration was measured in mice given intraperitoneal injections of glucose (1 g/kg of total body weight) or insulin (0.75 U/kg of total body weight) (mean ± SEM; UCP1-Cre *n =* 12 mice; p38α^UCP1-KO^ *n =* 14 mice). *p < 0.05; **p < 0.01; ***p < 0.001 UCP1-Cre versus p38α^UCP1-KO^ (2-way ANOVA coupled with Bonferroni’s post-tests or *t* test or Welch’s test when variances were different). See also S1 Data. BAT, brown adipose tissue; eWAT, epididymal fat; GTT, glucose tolerance test; HFD, high-fat diet; ITT, insulin tolerance test; iWAT, inguinal fat; pWAT, perirenal fat; sWAT, subcutaneous fat; UCP1, uncoupling protein 1; WAT, white adipose tissue.

In vitro–differentiated brown adipocytes from p38α^Fab-KO^ mice confirmed a key role of this kinase inhibiting browning in a cell-autonomous manner because several browning markers (UCP1, PGC1b, Cidea, Cox7a1, Cox7a2, and Cox8b) were up-regulated in p38α^Fab-KO^ brown adipocytes (S8A Fig). In concordance with the results observed in the BAT tissue, glycolytic genes were also up-regulated, while many lipogenic genes that correlated with the lower triglyceride content in p38α^Fab-KO^ brown adipocytes were down-regulated (S8B, S8C, S8D and S8E Fig). In addition, p38α^Fab-KO^ brown adipocytes have increased expression of perilipin with no changes in adiponectin, suggesting same differentiation capacity but smaller and more abundant lipid droplets (S8B Fig). On the other hand, p38α^Fab-KO^ white adipocytes presented the same in vitro differentiation rate judging by red-oil staining and the expression levels of adipocyte markers such as adiponectin and perilipin (S8F and S8G Fig). However, p38α^Fab-KO^ white adipocytes have increased expression of leptin (S8F Fig). To further confirm the autonomous role of p38α in BAT, we crossed p38α loxP mice with UCP1-Cre mice [24], which express Cre recombinase specifically in the interscapular brown fat at room temperature, generating p38α^UCP1-KO^ mice. In agreement with our previous results, these mice were protected against HFD-induced obesity and presented lower fat mass and increased temperature. Furthermore, they had lower blood glucose levels and partial glucose tolerance, indicating that they were protected against HFD-induced diabetes (Fig 4A–4F).

**Fig 5:**
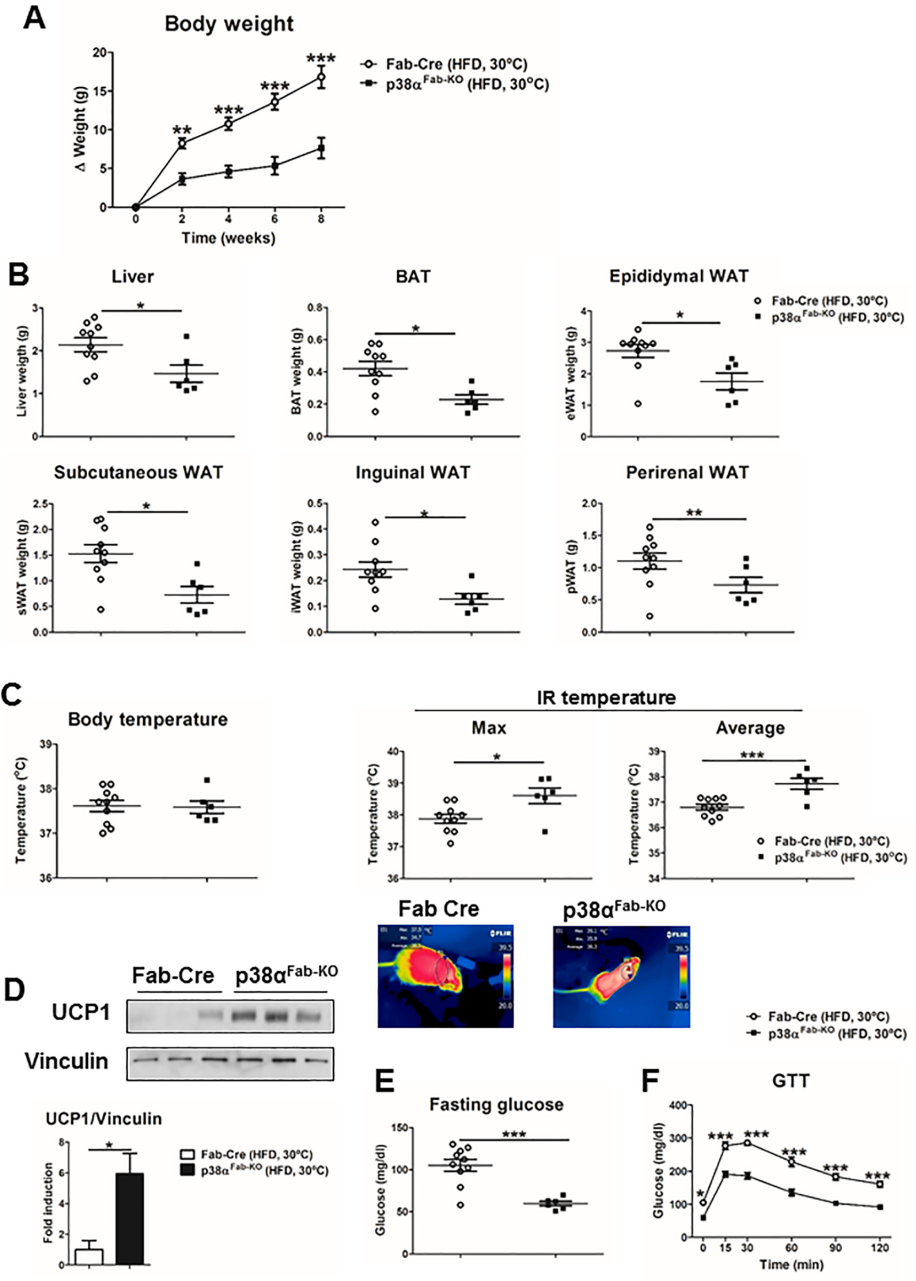
p38α^Fab-KO^ mice have increased BAT thermogenesis under thermoneutrality conditions. Fab-Cre and p38α^Fab-KO^ mice were fed an HFD for 8 weeks and housed at 30 °C. (A) Body weight time course in Fab-Cre and p38α^Fab-KO^ male (8–10-wk-old) mice fed a HFD over 8 weeks. Data are presented as the increase above initial weight (mean ± SEM; Fab-Cre *n =* 10 mice; p38α^Fab-KO^ *n =* 6 mice). (B) Weight of liver, BAT, eWAT, sWAT, iWAT, and pWAT (mean ± SEM; Fab-Cre *n =* 10 mice; p38α^Fab-KO^ *n =* 6 mice). (C) Body and skin temperature of surrounding interscapular BAT from HFD-fed Fab-Cre and p38α^Fab-KO^ mice (mean ± SEM; Fab-Cre *n =* 10 mice; p38α^Fab-KO^ *n =* 6 mice). Lower panels show representative infrared thermal images. (D) Immunoblot analysis of UCP1 protein levels in lysates from BAT. Quantification is shown in the lower panel. (E) Fasting and fed blood glucose in Fab-Cre and p38α^Fab-KO^ mice fed the HFD at 30 °C (mean ± SEM; Fab-Cre *n =* 10 mice; p38α^Fab-KO^ *n =* 6 mice). (F) GTT in HFD-fed Fab-Cre and p38α^Fab-KO^ at 30 °C. Blood glucose concentration was measured in mice given intraperitoneal injections of glucose (1 g/kg of total body weight) (mean ± SEM; Fab-Cre *n =* 10 mice; p38α^Fab-KO^ *n =* 6 mice). Statistically significant differences between Fab-Cre and p38α^Fab-KO^ mice are indicated: *p < 0.05; **p < 0.01; ***p < 0.001 (*t* test or Welch’s test when variances were different). See also S1 Data. BAT, brown adipose tissue; eWAT, epididymal fat; GTT, glucose tolerance test; HFD, high-fat diet; IR temperature, infrared temperature; iWAT, inguinal fat; pWAT, perirenal fat; sWAT, subcutaneous fat; UCP1, uncoupling protein 1; WAT, white adipose tissue.

Our data at 23 °C demonstrated that lack of p38α resulted in increased whole-body energy expenditure due to the activation of BAT and iWAT thermogenesis. At this temperature, BAT is already fully differentiated; because it is complicated to detect an even higher level of UCP1, genetic modifications that up-regulate UCP1 levels cannot be easily detected [25]. For this reason, we therefore evaluated p38α^Fab-KO^ phenotype in thermoneutrality (30 °C) because it has been suggested to be more similar to the human situation [25]. At 30 °C, p38α^Fab-KO^ mice were also protected against HFD-induced obesity (Fig 5A) and presented lower body fat mass and increased BAT thermogenesis (Fig 5B and 5C), indicating that, even at temperatures at which BAT is impeded, these mice maintain BAT activation. In fact, UCP1 expression was much higher in BAT from p38α^Fab-KO^ than in the control Fab-Cre mice at 30 °C (Fig 5D). In addition, p38α^Fab-KO^ were also protected from HFD-induced diabetes at thermoneutrality (Fig 5E and 5F). Together, these data confirm that lack of p38α protects against HFD-induced obesity and diabetes due to an activation of BAT thermogenesis.

**Fig 6:**
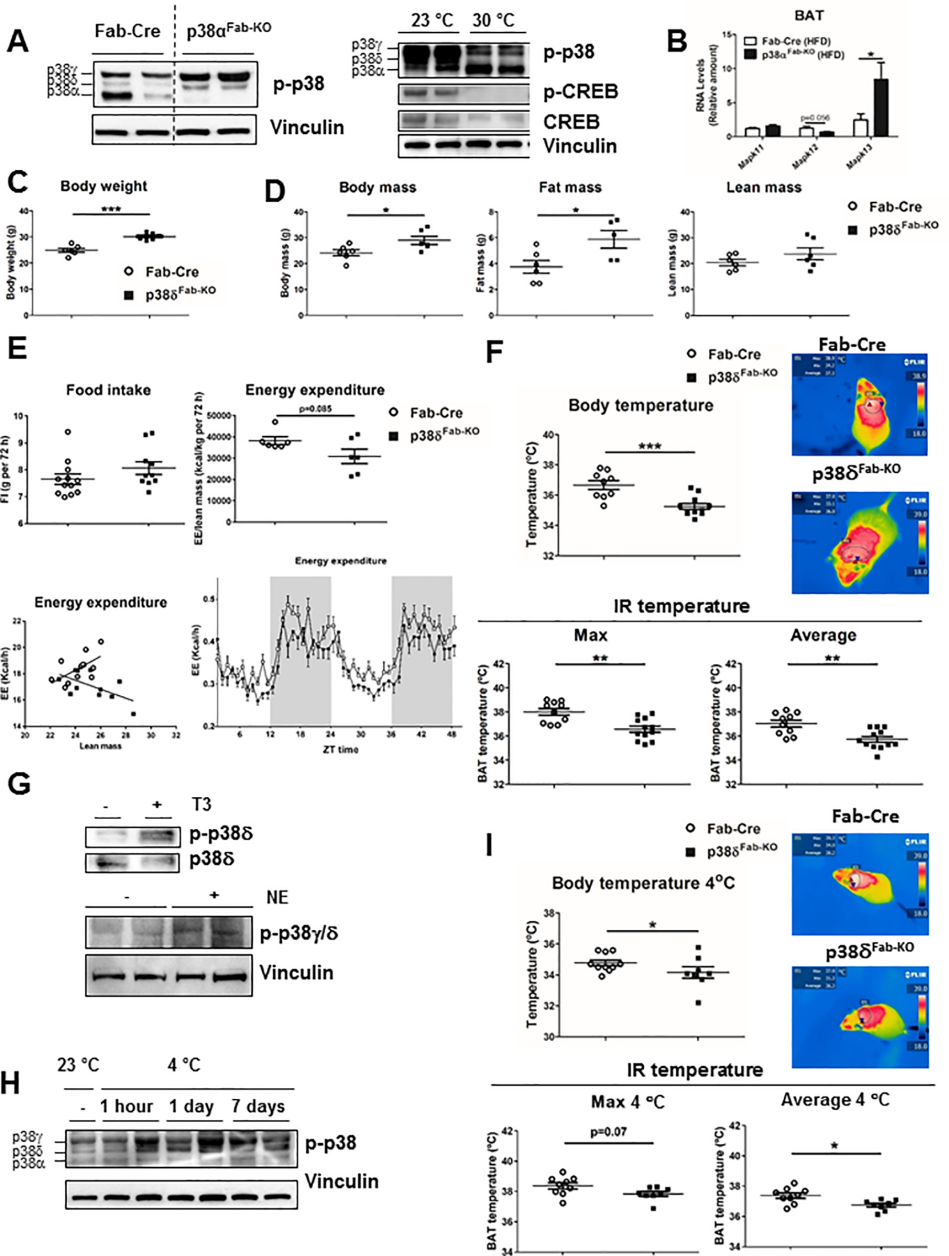
Activation of p38δ is responsible for BAT activation. (A) Immunoblot analysis of BAT lysate from Fab-Cre and p38α^Fab-KO^ mice fed an HFD for 8 weeks. Left: 23 °C; right: comparison of 23 °C versus 30 °C. (B) qRT-PCR analysis of mRNA expression of p38β (*Mapk11*), p38γ (*Mapk12*), and p38δ (*Mapk13*) in BAT Fab-Cre and p38α^Fab-KO^ mice fed an HFD for 8 weeks. mRNA was normalised to level of *Gapdh* mRNA (mean ± SEM, Fab-Cre *n =* 15 mice; p38α^Fab-KO^ *n =* 9 mice) (C) Body weight in Fab-Cre and p38δ^Fab-KO^ male (8–10-wk-old) mice fed an ND over 8 weeks (mean ± SEM; Fab-Cre *n =* 6 mice; p38δ^Fab-KO^ *n =* 6 mice). (D) Body, fat, and lean mass in p38δ^Fab-KO^ and Fab-Cre mice after 8 weeks of ND measured by NMR (mean ± SEM; Fab-Cre *n =* 6 mice; p38δ^Fab-KO^ *n =* 5 mice). (E) Comparison of energy balance between ND-fed Fab-Cre and p38δ^Fab-KO^ mice. ND-fed mice were examined in a metabolic cage over a 3-day period to measure FI and EE. FI (upper left panel; mean ± SEM; Fab-Cre *n =* 12 mice; p38δ^Fab-KO^ *n =* 10 mice) and EE (upper right panel; mean ± SEM; Fab-Cre *n =* 6 mice; p38δ^Fab-KO^ *n =* 6 mice) over 2 days were corrected by lean mass. EE expressed as ANCOVA analysis (lower left panel; mean ± SEM; Fab-Cre *n =* 9 mice; p38δ^Fab-KO^ *n =* 12 mice) and hour by hour over a 48-hour period (lower right panel; mean ± SEM; Fab-Cre *n =* 12 mice; p38δ^Fab-KO^ *n =* 12 mice) are also shown. (F) Body temperature of ND-fed Fab-Cre and p38δ^Fab-KO^ mice (mean ± SEM; Fab-Cre *n =* 9 mice; p38δ^Fab-KO^ *n =* 11 mice). Skin temperature surrounding interscapular BAT in ND-fed Fab-Cre and p38δ^Fab-KO^. Right panels show representative infrared thermal images (mean ± SEM; Fab-Cre *n =* 10 mice; p38δ^Fab-KO^ *n =* 12 mice). (G) Adipocytes differentiated from interscapular BAT were stimulated with 100 nM T3 for 48 hours. Immunoprecipitation from cell lysates of p38δ were evaluated by immunoblot with antibodies against phospho-p38 and p38δ. Adipocytes differentiated from sWAT were stimulated with 1 μM NE for 1 hour, and p38 phosphorylation was analysed by immunoblot. (H) Control mice (C57BL/6) were exposed to cold (4 °C) for the indicated time, and phosphorylation of the different p38s in BAT was evaluated by immunoblot (*n =* 5 for each group; representative blot presented). (I) Body temperature of ND-fed Fab-Cre and p38δ^Fab-KO^ mice exposed to cold (4 °C) for 1 hour (mean ± SEM; Fab-Cre *n =* 10 mice; p38δ^Fab-KO^ *n =* 8 mice). Skin temperature surrounding interscapular BAT in ND-fed Fab-Cre and p38δ^Fab-KO^ after 1 hour of cold exposure. Right panels show representative infrared thermal images (mean ± SEM; Fab-Cre *n =* 9 mice; p38δ^Fab-KO^ *n =* 8 mice). *p < 0.05; **p < 0.01; ***p < 0.001 (*t* test). See also S1 Data. BAT, brown adipose tissue; Creb, cAMP response element-binding; EE, energy expenditure; FI, food intake; HFD, high-fat diet; IR temperature, infrared temperature; ND, normal-chow diet; NE, norepinephrine; NMR, nuclear magnetic resonance; qRT-PCR, quantitative real-time polymerase chain reaction; sWAT, subcutaneous fat.

To gain insight into the molecular mechanism that might account for increased UCP1 levels and thermogenic capacity, we studied the signalling in the different adipose tissue depots. The p38MAPK pathway has been shown to trigger BAT activation in several models [18, 26–28]. Additionally, it has been found that p38α can inhibit the other p38 isoforms by a negative feedback loop that blocks the activation of the upstream kinases of this pathway [29]. Therefore, we evaluated the expression and phosphorylation state of the other p38s, with a phospho-p38 antibody that recognises all p38 isoforms [30]. Using adipocytes lacking p38γ/δ, we confirmed that p38α/β run around 38 kDa, while p38γ/δ run higher—around 41 kDa—allowing us to distinguish the phosphorylation of these kinases (S9A Fig). Under ND condition, p38δ and p38γ were hyperactivated in eWAT and iWAT from p38α^Fab-KO^ (S9B Fig). In agreement, p38δ/γ were activated more when cells were treated with sorbitol and p38α inhibitor SB203580 (S9C Fig). HFD resulted in reduced RNA expression of all the p38 isoforms in BAT, while in eWAT, only p38δ and p38γ decreased (S9D Fig). p38δ and p38γ were hyperactivated in iWAT and BAT from HFD-fed p38α^Fab-KO^, whereas elevated p38δ (*Mapk13*) RNA levels were also found in BAT and eWAT from HFD-fed p38α^Fab-KO^ animals (Fig 6A, 6B and S7, S9E and S9F Figs). Activation of p38δ in BAT was diminished when mice were maintained at 30 °C (Fig 6A), suggesting that this p38 isoform might activate BAT thermogenesis. To further evaluate this hypothesis, mice lacking p38δ in adipose tissue (p38δ^Fab-KO^) were generated. In agreement with the importance of this kinase in BAT activation, p38δ^Fab-KO^ mice fed with ND presented higher body weight, associated with increased fat mass and weight of all fat depots (Fig 6C and 6D and S10A Fig). In concordance, p38δ^Fab-KO^ presented reduced energy expenditure, whole-body temperature, and decreased BAT thermogenesis (Fig 6E and 6F) as well as lower expression levels of *Ucp1* and *Ppargc1*β in BAT (S10B Fig) with no differences in protein kinase A (PKA) phosphorylation (S10C Fig).

p38δ is activated in BAT upon cold exposure and in adipocytes after stimulation with the thyroid hormone T3 or norepinephrine (NE) (Fig 6G and 6H), suggesting that this p38 isoform might activate BAT thermogenesis. In fact, at 4 °C, p38δ^Fab-KO^ mice have lower body and skin temperature in the BAT region (Fig 6I). Moreover, HFD-fed p38δ^Fab-KO^ mice were more obese with higher fat mass and weight of all fat depots (S11A–S11C Fig). This increased adiposity correlated with lower BAT thermogenesis and lower UCP1, *Ppargc1a*, and *Cidea* levels in BAT (S11D–S11F Fig).

**Fig 7:**
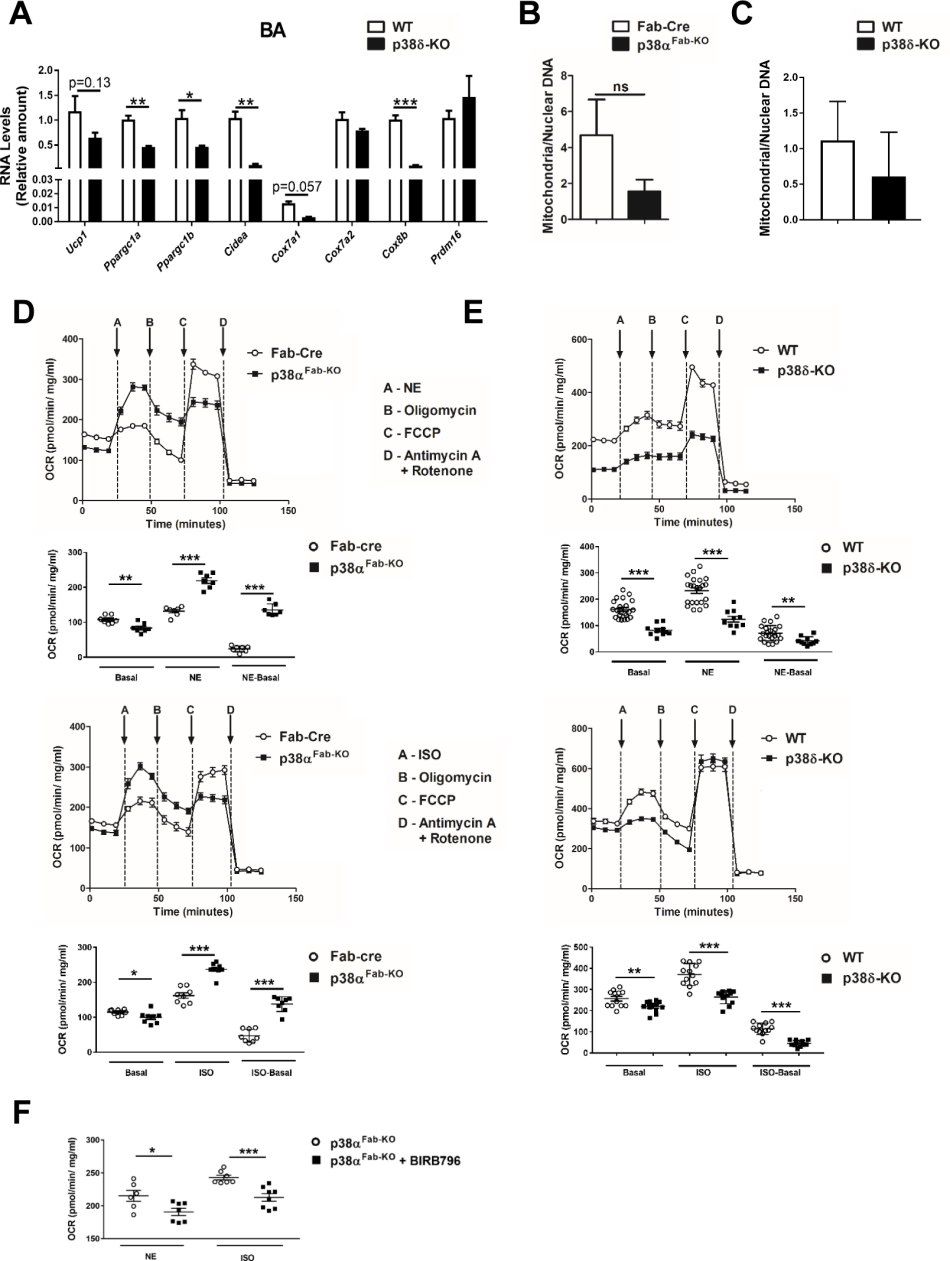
p38s regulate respiratory capacity of brown adipocytes. Primary adipocytes isolated from intercapsular BAT were differentiated in vitro. (A) qRT-PCR analysis of browning genes mRNA expression from primary adipocytes isolated from WT or p38δ^−/−^ mice. mRNA expression was normalised to the amount of *Gapdh* mRNA (mean ± SEM; WT *n =* 5 wells; p38δ^−/−^ *n =* 5 wells). (B) Analysis of mitochondrial DNA content with respect to nuclear DNA by RT-PCR in adipocytes isolated from BAT of Fab-cre or p38α^Fab-KO^ mice (mean ± SEM; Fab-Cre *n =* 3 wells; p38α^Fab-KO^ *n =* 5 wells) and of (C) WT or p38δ^−/−^ mice (mean ± SEM; WT *n =* 3 wells; p38δ^−/−^ *n =* 4 wells). (D–E) OCR to NE (1 μM) and ISO (1 μM) in differentiated brown adipocytes from Fab-Cre and p38α^Fab-KO^ mice (mean ± SEM; Fab-Cre *n =* 7 or p38α^Fab-KO^ *n =* 7 wells treated with NE; and Fab-Cre *n =* 8 or p38α^Fab-KO^ *n =* 8 wells treated with ISO) (panel D) or from WT or p38δ^−/−^ mice (mean ± SEM; WT *n =* 22 or p38δ^−/−^ *n =* 12 wells treated with NE; and WT *n =* 12 or p38δ^−/−^ *n =* 12 wells treated with ISO) (panel E) analysed by Seahorse assay. Nonmitochondrial respiration was subtracted from OCR values, and all values were normalised to protein content. Upper panels show OCR over time upon different drugs injections: oligomycin (1 μM), FCCP (1 μM), and antimycin A (1 μM) with rotenone (1 μM). Lower panels show basal and NE/ISO-induced OCR. (F) OCR induced by NE and ISO in differentiated brown adipocytes from Fab-Cre and p38α^Fab-KO^ mice was abolished by pretreatment with BIRB796 (10 μM) for 1 hour (mean ± SEM; Fab-Cre *n =* 6 or p38α^Fab-KO^ *n =* 7 wells treated with NE; and Fab-Cre *n =* 7 or p38α^Fab-KO^ *n =* 8 wells treated with ISO). See also S1 Data. BAT, brown adipose tissue; FCCP, carbonyl cyanide-4-(trifluoromethoxy)phenylhydrazone; ISO, isoproterenol; NE, norepinephrine; OCR, oxygen consumption rate; qRT-PCR, quantitative real-time polymerase chain reaction; WT, wild-type.

Our data indicated that p38δ was triggering thermogenesis because in vitro–differentiated brown adipocytes lacking p38δ have reduced expression of important genes implicated in BAT thermogenesis (*Ppargc1b*, *Ppargc1a*, *Cidea*, and *Cox8b*) and a slight decrease of *Ucp1* and *Cox7a1* supporting the cell-autonomous effect of p38δ in BAT thermogenesis (Fig 7A), with no differences in amount of mitochondrial DNA (Fig 7B and 7C).

Therefore, we evaluated respiration profiles in brown adipocytes lacking p38α and p38δ. Brown adipocytes lacking p38α presented higher leak respiration after isoproterenol (ISO) or NE treatment (Fig 7D). However, this augmented respiration capacity induced by NE or ISO was diminished when p38δ was chemically inhibited by BIRB796, a known inhibitor p38δ [31], as well as in p38δ-deficient brown adipocytes (Fig 7E and 7F), supporting the important role of this kinase in brown adipocyte activation.

**Fig 8:**
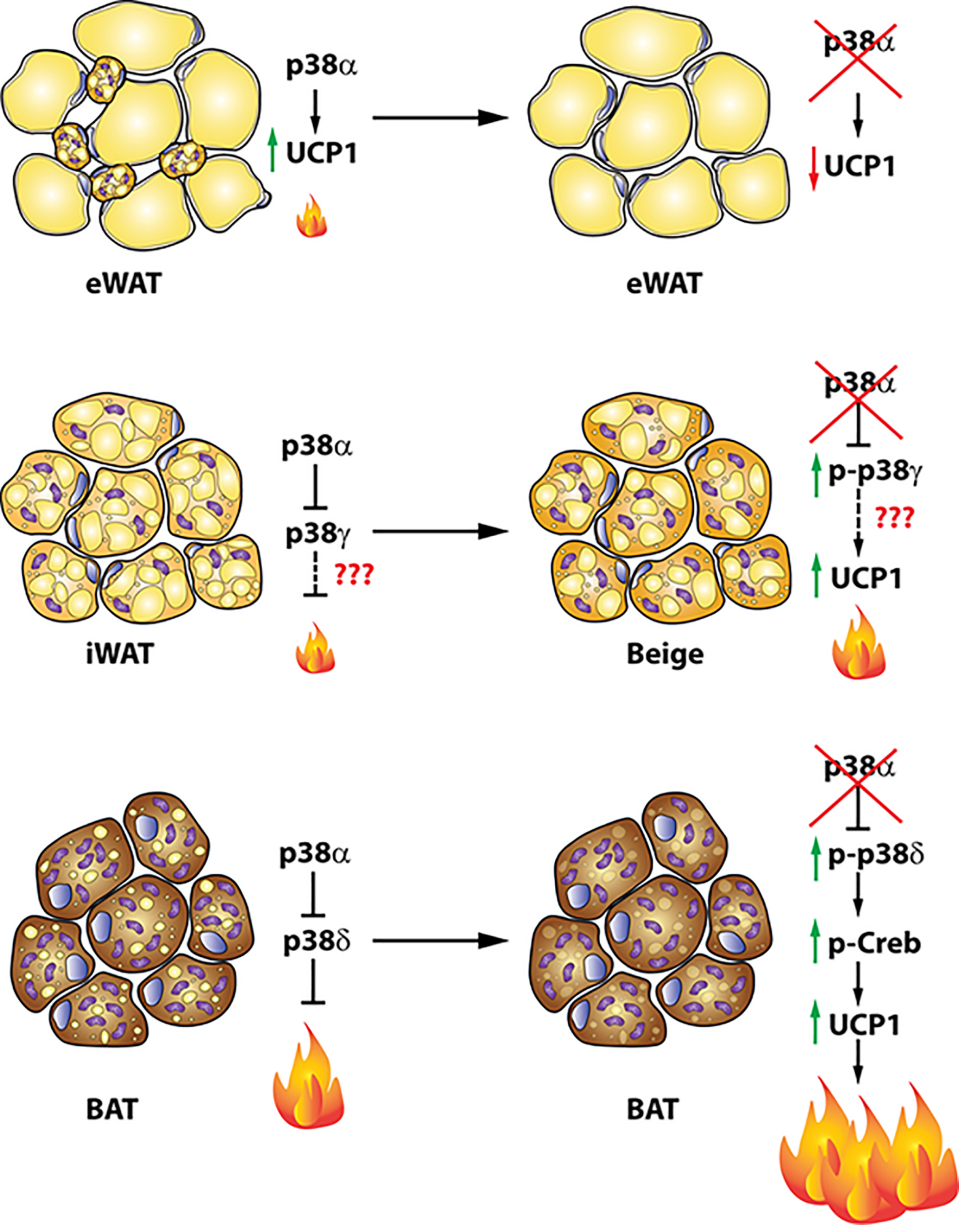
Regulation of browning and BAT activation by p38 pathway. Graphical abstract summarising the role of p38 isoforms in adipose tissue. In eWAT, p38α activates browning through the phosphorylation of Creb and ATF2 increasing UCP1 expression. In iWAT and BAT, p38α activation inhibits p38γ and p38δ and in consequence reduces browning and BAT activation, respectively, by down-regulation of UCP1. ATF2, activating transcription factor 2; BAT, brown adipose tissue; Creb, cAMP response element-binding; eWAT, epididymal fat; iWAT, inguinal fat; UCP1, uncoupling protein 1.

In conclusion, we demonstrated that p38α in BAT inhibits p38δ activation, which in turn regulates BAT thermogenesis, energy expenditure, and body weight. We demonstrated that p38α and p38δ have opposite roles in BAT: whereas p38α inhibits BAT thermogenesis, p38δ induces it upon several physiological stimuli (Fig 8).

## Discussion

Adipose tissue has become an important target for the treatment of obesity, not only because its dysfunction could be responsible for diabetes development but also because increasing BAT thermogenesis and/or browning of WAT could lead to new therapeutic approaches against obesity [32, 33]. In this scenario, p38MAPK signalling has been proposed to be a key activator of these processes. Consequently, there is an increasing interest to understand the function of this pathway in the regulation of adipose tissue metabolism, remodelling, and browning.

A growing number of studies have defined p38MAPK as one of the main pathways that stimulates browning and BAT thermogenesis [18,21–23]. However, using genetically modified mice lacking specific p38 family members in adipose tissue, we have shown that lack of p38α in adipose tissue protects against HFD-induced obesity by increasing energy expenditure through the activation of BAT thermogenesis. Mechanistically, lack of p38α results in hyperactivation of p38δ in BAT together with increased UCP1 expression and higher Creb and AMPK phosphorylation. Negative feedback controls by p38α through the regulation of upstream activators of the pathway—such as TAB1 phosphorylation or MKK6 expression—have been previously reported [29, 34]. Here, we show that negative control of the pathway by p38α has biological and pathological implications. However, it would be interesting to examine the epistatic relationship between p38α and p38δ genetically in future studies.

We also demonstrated that p38δ is activated in BAT by 3 stimuli widely known to activate this tissue: cold exposure, NE, and thyroid hormone treatment [35, 36], whereas its phosphorylation is reduced under thermoneutrality conditions. In addition, p38δ expression in BAT was reduced in obese mice, while this down-regulation was ablated in p38α^Fab-KO^ mice, suggesting that activation of p38δ in p38α^Fab-KO^ mice is responsible for the protection against diet-induced obesity observed in these mice. Indeed, inhibition of p38δ in p38α^Fab-KO^ brown adipocytes abolished the increased respiratory capacity induced by β3-adrenergic stimuli. In agreement with the role of p38δ-promoting thermogenesis, mice lacking this kinase in adipose tissue developed overweight, even in ND, and showed decreased whole-body energy expenditure associated with lower temperature and reduced BAT activation. Moreover, we confirmed the cell-autonomous role of p38δ inducing browning using differentiated adipocytes.

Our results were completely unexpected because the p38MAPK pathway has been shown to trigger BAT activation in several models [18, 26–28], and—until now—it was thought that the only implicated family member was p38α. Moreover, we have recently found that hyperactivation of p38α in MKK6-deficient animals induces browning of eWAT [37]. These finding might indicate opposite effects of p38α in eWAT versus iWAT or BAT. While p38α would activate browning in eWAT—increasing energy expenditure—it would prevent it in iWAT, and it would block thermogenesis through the negative regulation of p38δ in BAT. In agreement with this hypothesis, we observed reduced levels of UCP1 in epididymal fat lacking p38α. In fact, our data from human samples indicated that the p38α mRNA levels in visceral fat directly correlates with UCP1 expression and inversely correlates with the BMI, suggesting that p38α triggers visceral fat browning. We also found that p38α in sWAT inversely correlates with UCP1. This is in accordance with results observed in mouse models, in which we found a decrease of all p38s after HFD in all fat depots. However, the levels of UCP1 expression in these human fat depots is quite low judging by the low Ct obtained (higher than 29), and evaluation of UCP1 protein expression in human fat depots would be necessary. Moreover, further studies to determinate the expression of p38 family members and upstream kinases in other human fat depots would help us to understand the role of these kinases in human adipocytes.

It has been proposed that p38α induces adipogenesis [38–40]. However, using genetically modified animals, we showed here that lack of p38α in preadipocytes did not affect their differentiation to adipocytes, nor did it affect changes in the differentiation markers evaluated in the major fat depots. This capacity of cells lacking p38α to still differentiate to adipocytes could be due to the hyperactivation of the other members of the family: p38γ and p38δ. In fact, it has been shown that p38 isoforms can compensate for each other [30]. Here, we demonstrated the cell-autonomous and opposite effects of 2 p38 isoforms in adipocytes, p38α and p38δ. The cell-specific actions of p38α in each fat depot could be explained by the specific expression pattern of p38 family members—p38α being the main isoform expressed in eWAT, whereas p38δ or p38γ are abundant in BAT or iWAT. Furthermore, our results suggest a different regulation of p38s expression in adipose tissue during obesity, with only decrease of p38δ and p38γ in eWAT and no effects in p38α or p38β. More studies would be necessary to elucidate the function of p38γ in adipose tissue.

We also evaluated the controversial role of p38α in GLUT4 translocation [41–43]. Under ND, insulin-induced GLUT4 translocation was the same in both control and p38α^Fab-KO^ mice. However, p38α^Fab-KO^ mice maintained the insulin-induced translocation after the HFD, perhaps due to the fact that these animals did not gain weight and were protected against diet-induced insulin resistance. In fact, our data suggest that these mice are more glucose tolerant using a dose of glucose based on their total body weight.

Due to the potential clinical implications of these results, it would be necessary to further evaluate the function of each p38 family member in browning to better understand how this pathway controls adipose tissue metabolism.

In summary, we have demonstrated that p38α and p38δ in adipose tissue have opposite roles: p38α negatively regulates BAT thermogenesis, energy expenditure, and body weight, while p38δ induces thermogenesis in BAT in response to several physiological stimuli. These results have potential clinical implications because inhibition of p38α or activation of p38δ might be of therapeutic interest against obesity.

## Material and methods

### Ethics statement

This population study was approved by the Ethics Committee of the University Hospital of Salamanca and the Carlos III (CEI PI 09_2017-v3) with the all subjects providing written informed consent to undergo visceral fat biopsy under direct vision during surgery. Data were collected on demographic information (age, sex, and ethnicity), anthropomorphic measurements (BMI), smoking and alcohol history, coexisting medical conditions, and medication use.

All animal procedures conformed to EU Directive 86/609/EEC and Recommendation 2007/526/EC regarding the protection of animals used for experimental and other scientific purposes, enacted under Spanish law 1201/2005. The protocols are CNIC 08/13 and PROEX 49/13.

### Study population and sample collection

For the analysis of visceral fat, the study population included 71 patients (58 adult patients with BMI ≥35), while for the analysis of sWAT, the study population included 170 patients (140 adult patients with BMI ≥35), recruited from patients who underwent elective bariatric surgery at the University Hospital of Salamanca. Patients were excluded if they had a history of alcohol use disorders or excessive alcohol consumption (>30 g/day in men and >20 g/day in women) or had chronic hepatitis C or B. Control subjects (*n* = 13 for visceral fat study; *n* = 30 for sWAT study) were recruited among patients who underwent laparoscopic cholecystectomy for gallstone disease. Before surgery, fasting venous blood samples were collected for measuring complete cell blood count, total bilirubin, aspartate aminotransferase (AST), alanine aminotransferase (ALT), total cholesterol, high-density lipoprotein, low-density lipoprotein, triglycerides, creatinine, glucose, and albumin (S1 and S2 Tables).

### Animal models

Mice with a germ-line mutation in *Mapk14* (*p38*α) and *Mapk13* (*p38*δ) have been reported before [44, 45]. These animals were crossed with Tg (Fabp4-cre)1Rev/J [46] line or B6.FVB-Tg(Ucp1-cre)1Evdr/J [24] on the C57BL/6J background (Jackson Laboratory) to generate the mice lacking p38α or p38δ in adipose tissue (both WAT and BAT or just in BAT, respectively). All mice were maintained on a C57BL/6J background (back-crossed 10 generations). Genotype was confirmed by PCR analysis of genomic DNA. Mice were fed with an ND or an HFD, Research Diets Inc.) for 8 weeks ad libitum. For fat expansion measurement, mice were treated with BrdU (0.4 mg/ml; Sigma) in the drinking water (water was refreshed every 3 days) during the first week of a 6-week HFD. For temperature experiments, mice were housed at 30 °C for 8 weeks while feeding an HFD in case of thermoneutrality analysis. Mice were exposed to 4 °C for 1 hour, 1 day, or 1 week in case of cold adaptation studies.

### Cell culture

Immortalised and primary brown preadipocytes from WT, Fab-Cre, p38α^Fab-KO^, and p38δ-KO mice were differentiated to brown adipocytes in 10% FCS medium supplemented with 20 nM insulin, 1 nM T3, 125 μM indomethacin, 2 μg/ml dexamethasone, and 50 mM IBMX for 48 hours and maintained with 20 nM of insulin and 1 nM of T3 for 8 days. For some experiments, cultures were incubated with 100 nM T3 for 48 hours before extraction.

Immortalised white preadipocytes from Fab-Cre and p38α^Fab-KO^ mice were differentiated to adipocytes for 9 days in 8% FCS medium supplemented with 5 μg/ml insulin, 25 μg/ml IBMX, 1 μg/ml dexamethasone, and 1 μM troglitazone. For some experiments, cultures were incubated with 1 μM NE for 1 hour before extraction.

### Analysis of mitochondrial function

Primary brown preadipocytes were plated and differentiated in gelatin-coated (0.1%) 96 seahorse plates. MitoStress oxygen consumption rate (OCR) was assessed in XF medium containing 25 mM glucose, 2 mM L-glutamine, and 1 mM sodium pyruvate using a XF-96 Extracellular Flux Analyzers (Seahorse Bioscience, Agilent Technologies). Cells were stimulated with following drugs: NE or ISO, oligomycin, FCCP, and antimycin A plus rotenone (1 μM finally; all from Sigma Aldrich). The protocol for the all drugs followed a 3-minute mix, 2-minute wait, and 3-minute measure cycle that was repeated 3 times. After the analysis, data were normalised to protein level assessed by Bradford quantification. Basal Respiration Capacity (OCR basal − OCR nonmitochondrial) and oxygen consumption in response to NE (OCR NE − OCR basal) or ISO (OCR ISO–OCR basal) were calculated. For some experiments, cultures were pretreated with 10 μM BIRB796 for 1 hour.

### Western blot

Samples were lysed with RIPA buffer containing protease and phosphatase inhibitors (Tris-Hcl 50 mM [pH 7.5]; Triton X-100 1%; EDTA 1 mM [pH 8]; EGTA 1 mM; NaF 50 mM; β-glycerophosphate-Na 1 mM; sodium pirophosphate 5 mM; orthovanadate-Na 1 mM; sucrose 0.27 M; PMSF 0.1 mM; β-mercaptoethanol 1 mM; aprotinin 10 μg/ml; leupeptin 5 μg/ml). Lysates were separated by SDS-PAGE and incubated with antibodies diluted 1/1,000 against P-Akt308 (Cell Signaling, 9275s), P-Akt473 (Cell Signaling, 9271s), Akt (Cell Signaling, 9272s), UCP1 (Abcam, AB10983), P-ATF2 (Cell Signaling, 9225s), ATF2 (Cell Signaling, 9226s), P-CREB (Cell Signaling, 9198), CREB (Cell Signaling, 4820s), P-p38 (Cell Signaling, 9211s)—which recognises the phosphorylation in the activation sites of all the p38 isoforms—p38α (Santa Cruz, sc-535), P-AMPKα (Cell Signaling, 2531s), AMPKα (Cell Signaling, 2603s), P-ACC (Cell Signaling, 3661s), ACC (Cell Signaling, 3676s), PGC1α (Santa Cruz, sc13067), GAPDH (Santa Cruz, sc25778), tubulin (Sigma, T6199), and vinculin (Sigma, V9131), followed by an incubation with a secondary antibody conjugated with HRP. Reactive bands were detected by chemiluminescence and quantified by Image J software. Specificity of UCP1 antibody was evaluated using brown and eWAT from UCP1 KO animals [47].

For the immunoprecipitation assay, cell extracts were incubated with 4 μg of anti-p38 delta coupled with protein-G-Sepharose. After an overnight incubation at 4°C, the captured proteins were centrifuged at 10,000 g, the supernatants discarded, and the beads washed 4 times in lysis buffer. Beads were boiled for 5 minutes at 95 °C in 10 μl sample buffer. The antibodies employed were anti-phospho p38 and anti-p38δ (Santa Cruz, sc7585). Immune complexes were detected by enhanced chemiluminescence (NEN).

### Fluorescence-assisted cell sorting

Mouse bone marrow (BM) and spleens were collected, and single-cell suspension was obtained. Erythrocytes were lysed with a red cell lysis buffer incubation for 3 minutes on ice. Spleen samples were enriched using CD3 (BioLegend 79751 clone 145-2C11) and B220 (BioLegend 79752 clone RA3-6B2) biotinylated antibodies and magnetic Dynabeads Myone streptavidin T1 (invitrogen). Myeloid cells from spleen were labelled by surface staining with FITC-conjugated CD11b (BioLegend 79749 clone M1/70), PE-conjugated Gr1 (Ly6G/Ly6C) (BDBioscience 79750 clone RB6-8C5), and APC-conjugated F4/80 (eBiosciences 25-4801-82 clone BM8) antibodies, and myeloid cells from BM were labelled by FITC-conjugated Gr1 (Ly6G/Ly6C) (Invitrogen 11-5931-82 clone RB6-8C5), PE-conjugated CD115 (eBioscience 12-1152-82 clone AFS98), and APC-conjugated F4/80 (eBiosciences 25-4801-82 clone BM8) antibodies. Nuclei were stained with DAPI. Cells were sorted with a fluorescence-assisted cell sorting (FACS) Aria (BD) as follows: spleen macrophages (Gr1^−^ Cd11b^medium^ F4/80^+^), spleen neutrophils (Gr1^high^ Cd11b^+^), and BM monocytes (CD115^+^ F4/80^−^). Isolated myeloid cells were lysed and analysed by western blot.

### GTT

Overnight-starved mice were injected intraperitoneally with 1 g/kg of body weight of glucose, and blood glucose levels were quantified with an Ascensia Breeze 2 glucose meter at 0, 15, 30, 60, 90, and 120 minutes post injection. Alternatively, GTT was performed injecting intraperitoneally 1 g/kg of lean mass of glucose.

### ITT

ITT was performed by injecting intraperitoneally 0.75 IU/kg of insulin at mice starved for 1 hour and detecting blood glucose levels with a glucometer at 0, 15, 30, 60, 90, and 120 minutes post injection.

### Indirect calorimetry system

Energy expenditure, respiratory exchange, and food intake were quantified using the indirect calorimetry system (TSE LabMaster, TSE Systems, Germany) for 3 days.

### Temperature

Body temperature was detected by a rectal thermometer (AZ 8851 K/J/T Handheld Digital Thermometer-Single, AZ Instruments Corp., Taiwan).

BAT-adjacent interscapular temperature was quantified by thermographic images using a FLIR T430sc Infrared Camera (FLIR Systems, Inc., Wilsonville, OR) and analysed through FlirIR software.

### Nuclear magnetic resonance analysis

Body, fat, and lean mass were quantified by nuclear magnetic resonance (Whole Body Composition Analyzer; EchoMRI, Houston, TX) and analysed by ImageJ software.

### Triglyceride measurement

Blood triglyceride content was quantified using a Dimension RxL Max analyser (Siemens). For triglyceride analysis in cells, brown adipocyte cultures were lysed in isopropanol, centrifuged at 10,000 g for 15 minutes at 4 °C, and triglycerides were detected in the supernatant with a commercial kit (Sigma).

### DNA isolation

Brown adipocyte cells were scraped in PBS and pellet lysed in TNES buffer supplemented with Proteinase K (20 mg/ml) overnight at 55 °C. Reaction was stopped with sodium chloride 6 M and samples centrifuged 5 minutes at 13,000 g. DNA was precipitated in supernatants with 100% ethanol and washed with 70% ethanol. After drying, DNA was resuspended in DNase free water, quantified, and analysed by RT-PCR. Mitochondrial DNA was detected using primers for COII and nuclear DNA, using primers for Sdh1 (S3 Table).

### qRT-PCR

RNA 500ng—extracted with RNeasy Plus Mini kit (Quiagen) following manufacturer instructions—was transcribed to cDNA, and qRT-PCR was performed using Fast Sybr Green probe (Applied Biosystems) and the appropriated primers in the 7900 Fast Real Time thermocycler (Applied Biosystems). Relative mRNA expression was normalised to *Gapdh* mRNA measured in each sample. Primers used are listed in S3 Table.

### Histology staining

Fresh livers, brown, and epididymal white fat were fixed with formalin 10%, included in paraffin, and cut in 5 μm slides followed by a haematoxylin–eosin staining.

Fat droplets were detected by oil red staining (0.7% in propylenglycol) in 8 mm slides included in OCT compound (Tissue-Tek) and in differentiated brown and white adipocytes.

### Immunostaining

Brown adipocytes were stained with Mito Tracker Deep Red (Invitrogen) and Bodipy (Invitrogen). Images were captured using Leica SPE confocal microcope (Leica Microsystems, Wetzlar, Germany).

For UCP1 immunostaining, brown and epididymal white fat were fixed with formalin 10%, included in paraffin, cut in 5 μm slides, and sequentially stained with a UCP1 antibody (1/500, Abcam, AB10983), a biotinylated goat anti-rabbit secondary antibody (1/500, Jackson Immuno Research Laboratories), a streptavidin-conjugated ABC complex (Vector Laboratories), and the substrate 3,3’-diaminobenzidene conjugated with horseradish peroxidase (Vector Laboratories), followed by brief counterstaining with Nuclear Fast Red haematoxylin (Sigma).

For immunofluorescence analysis, the 5 μm tissue sections were deparaffinised and rehydrated, followed by antigen retrieval in 10 mM sodium citrate (pH 6.0) under pressure in a CertoClav EL (CertoClav Sterilizer GmbH). For BrdU staining, sections were treated with DNase 30 minutes at 37 °C. Blocking and staining was performed in 5% BSA in PBS. Sections were incubated in primary antibodies including rat-anti-Ki67 (eBioscience, 14-5698-82; clone:SolA15) (1:100), rabbit-anti-GLUT4 (Abcam, ab654) (1:1000), mouse-anti-Caveolin-1 (Sigma, SAB4200216) (1:500), rat anti-BrdU (Abcam, Ab6326; clone: BU1/75 [ICR1]) (1:200), and rabbit anti-Perilipin (Cell Signaling, 9349; clone: D1D8) (1:400) overnight at 4 °C. Secondary antibodies including goat anti-rabbit-A488, goat anti-rat-A647, and chicken anti-mouse-A647—all used at 1:500—were purchased from Molecular Probes and incubated with tissue for 1 hour at room temperature. Nuclei were stained with DAPI, and slides were mounted with Vectashield mounting medium (Vector Laboratories) and examined using SP5 multi-line inverted confocal microscope. Several confocal images of each tissue section were acquired and analysed for the translocation of GLUT4 or the presence of Ki67 or BrdU in adipocyte nuclei. BAT and WAT cellularity were quantified using Fiji software. Adipocyte nuclei were identified by their location inside adipocyte membranes as described [23].

### Statistical analysis

Results are expressed as mean ± SEM. Statistical analysis was evaluated by student *t* test and 2-way ANOVA coupled with Bonferroni’s post-tests with values of *p* < 0.05 considered significant. When variances were different, Welch’s test was used. For human studies, variables were compared by means of Mann-Whitney U test or χ^2^ test.

## Supporting information

S1 Fig Deletion of p38α in white and BAT from p38α^Fab-KO^ mice, related to Fig 2. (a) Western blot analysis of p38α expression in BAT, eWAT, spleen, BM, and liver isolated from p38α^Fab-KO^ and control (Fab-Cre) mice. (b) Western blot analysis of p38α expression in BM, Mɸ, Neutros, and Mono. Mɸ and Neutros were sorted from spleen, and Mono from BM by FACS. BAT, brown adipose tissue; BM, bone marrow; eWAT, epididymal white adipose tissue; FACS, fluorescence assisted-cell sorting; Mɸ, macrophages; Mono, monocytes; Neutros, neutrophils.
(TIF)

S2 Fig Lower fat mass and improved glucose tolerance in ND-fed p38α^Fab-KO^ mice, related to Fig 2. (a) Body weight time course in Fab-Cre and p38α^Fab-KO^ male (8- to 10-week-old) mice fed an ND over 8 weeks. Data are presented as the increase above initial weight (mean ± SEM, Fab-Cre *n* = 9 mice; p38α^Fab-KO^ *n* = 8 mice). (b) NMR analysis of fat mass in p38α^Fab-KO^ and Fab-Cre mice after 8 weeks of ND (mean ± SEM, Fab-Cre *n* = 9 mice; p38α^Fab-KO^ *n* = 7 mice). (c) Weight of eWAT, pWAT, sWAT, iWAT, BAT, and liver relativized to tibia length (mean ± SEM, Fab-Cre *n* = 8 mice; p38α^Fab-KO^ *n* = 7 mice). (d) Fasting and fed blood glucose in Fab-Cre and p38α^Fab-KO^ mice fed an ND (8 weeks) (mean ± SEM, Fab-Cre *n* = 9 mice; p38α^Fab-KO^ *n* = 8 mice). (e) GTT and ITT in Fab-Cre and p38α^Fab-KO^ mice fed HFD for 8 weeks. Mice were fasted overnight (for GTT) or 1 hour (for ITT), and blood glucose concentration was measured in mice given intraperitoneal injections of glucose (1 g/kg of total body weight) or insulin (0.75 U/kg of total body weight). (mean ± SEM, Fab-Cre *n =* 9 mice; p38α^Fab-KO^ *n =* 8 mice). (f) Immunohistochemistry of eWAT sections using anti-GLUT4 (green), anti-Cav-1 (red) antibodies, and the nuclear dye DAPI (blue). Location of GLUT4 was analysed in mice treated with or without insulin (1.5 IU/kg) for 15 minutes after overnight fasting. Scale bar: 20 μm. (g) Comparison of energy balance between ND-fed Fab-Cre and p38α^Fab-KO^ mice. ND-fed mice were examined in a metabolic cage over a 3-day period to measure EE. EE levels corrected by lean mass (left panel), expressed as ANCOVA analysis (right panel) and hour by hour over a 48-hour period (lower panel) are shown (mean ± SEM, Fab-Cre *n =* 9 mice; p38α^Fab-KO^ *n =* 7 mice). (h) Body temperature of ND-fed Fab-Cre and p38α^Fab-KO^ mice (mean ± SEM, Fab-Cre *n =* 7 mice; p38α^Fab-KO^ *n =* 5 mice). *p < 0.05; **p < 0.01; ***p < 0.001 Fab-Cre versus p38α^Fab-KO^ (2-way ANOVA coupled with Bonferroni’s post-tests or *t* test or Welch’s test when variances were different). See also S1 Data. BAT, brown adipose tissue; Cav-1, caveolin-1; EE, energy expenditure; eWAT, epididymal fat; GLUT4, glucose transporter type 4; GTT, glucose tolerance test; HFD, high-fat diet; iWAT, inguinal fat; ITT, insulin tolerance test; ND, normal-chow diet; pWAT, perirenal fat; sWAT, subcutaneous fat.
(TIF)

S3 Fig ND-fed p38α^Fab-KO^ mice present increased expression of metabolic genes, related to Fig 2. (a) qRT-PCR analysis of mRNA expression of browning, adipogenic, glycolytic, β-oxidation and lipogenic genes from BAT of ND-fed Fab-Cre and p38α^Fab-KO^ mice. mRNA expression was normalized to the amount of *Gapdh* mRNA. (b) Immunoblot analysis of PGC1α protein levels in BAT of ND-fed Fab-Cre and p38α^Fab-KO^ mice (c) qRT-PCR analysis of mRNA expression of browning, adipogenic, glycolytic, β-oxidation and lipogenic genes from eWAT of ND-fed Fab-Cre and p38α^Fab-KO^ mice. mRNA expression was normalized to the amount of *Gapdh* mRNA (mean ± SEM, Fab-Cre *n =* 7 mice; p38α^Fab-KO^ *n =* 7 mice). *p < 0.05; **p < 0.01. Fab-Cre versus p38α^Fab-KO^ (*t* test or Welch’s test when variances were different). See also S1 Data. Acaca, acetyl-CoA carboxylase 1; Acox1, acyl-CoA oxidase 1; Adipoq, Adiponectin; BAT, brown adipose tissue; cidea, cell death activator; Cpt1a, carnitine palmitoyltransferase 1A; Cpt2, carnitine palmitoyltransferase 2; Dgat1, diacylglycerol acyltransferase-1; Dgat2, diacylglycerol acyltransferase-2; Elovl, fatty acid elongase 6; eWAT, epididymal fat; Fasn, fatty acid synthase; G6pc, glucose-6-phosphatase catalytic subunit; Glys2, glycogen synthase 2; ND, normal-chow diet; Pepck, phosphoenolpyruvate carboxykinase; PGC1α, proliferator-activated receptor gamma coactivator 1α; Plin1, perilipin 1; Ppard, peroxisome proliferator-activated receptor delta; Pparg, peroxisome proliferator-activated receptor gamma; Prdm16, PR domain zinc finger protein 16; qRT-PCR, quantitative real-time polymerase chain reaction; Scd1, stearoyl-CoA desaturase-1.
(TIF)

S4 Fig HFD-fed p38α^Fab-KO^ mice are protected against diet-induced diabetes, related to Fig 2. Fab-Cre and p38α^Fab-KO^ mice were fed an HFD for 8 weeks. (a) Weight of eWAT, sWAT, iWAT, pWAT, BAT, and liver relativized to tibia length (mean ± SEM, Fab-Cre *n =* 10 mice; p38α^Fab-KO^ *n =* 8 mice). (b) GTT in Fab-Cre and p38α^Fab-KO^ mice fed the HFD for 8 weeks. Mice were fasted overnight, and blood glucose concentration was measured in mice given intraperitoneal injections of glucose (1 g/kg of lean mass) (mean ± SEM, Fab-Cre *n =* 5 mice; p38α^Fab-KO^ *n =* 6 mice). (c) Western blot analysis of Akt activation in the liver from Fab-Cre mice fed with ND or HFD. Mice were treated without or with insulin (1.5 IU/kg) for 15 minutes after overnight fasting. Each line represents a different mouse. (d) Western blot analysis of Akt activation in the liver, skeletal muscle, eWAT, and BAT from mice fed with HFD. Mice were treated without or with insulin (1.5 IU/kg) for 15 minutes after overnight fasting. Each line represents a different mouse. (e) Triglyceride content in blood samples from Fab-Cre and p38α^Fab-KO^ mice (mean ± SEM, Fab-Cre *n =* 12 mice; p38α^Fab-KO^ *n =* 8 mice). *p < 0.05, ***p < 0.001 Fab-Cre versus p38α^Fab-KO^ (2-way ANOVA coupled with Bonferroni’s post-tests or *t* test or Welch’s test when variances were different). See also S1 Data. BAT, brown adipose tissue; eWAT, epididymal white fat; GTT, glucose tolerance test; Fts, Fasted; HFD, high-fat diet; iWAT, inguinal fat; ND, normal-chow diet; pWAT, perirenal WAT; sWAT, subcutaneous fat.
(TIF)

S5 Fig Fat depots from HFD-fed p38α^Fab-KO^ mice present smaller adipocytes, related to Fig 3. Fab-Cre and p38α^Fab-KO^ mice were fed an HFD for 8 weeks. (a) Immunohistochemistry of eWAT sections using anti-Ki67 (red), anti-perilipin (green) antibodies, and the nuclear dye DAPI (blue) (upper panel). Scale bar: 20 μm. A positive cell is shown in a bigger magnification for each genotype. Quantification of proliferation and adipocyte size are shown (lower panel) (mean ± SEM, Fab-Cre *n =* 5 mice; p38α^Fab-KO^ *n =* 5 mice and 5 pictures of each mouse). (b) Staining of UCP1 after 8 weeks of HFD in eWAT. Representative pictures are shown from Fab-Cre *n =* 6 mice; p38α^Fab-KO^ *n =* 6 mice with 3 pictures of each mouse. Scale bar: 50 μm. (c) Immunohistochemistry of BAT sections using anti-Ki67 (red), anti-perilipin (green) antibodies, and the nuclear dye DAPI (blue) (upper panel). Scale bar: 20 μm. A positive cell is shown in a bigger magnification for each genotype. Quantification of proliferation and adipocyte size are shown (lower panel) (mean ± SEM, Fab-Cre *n =* 5 mice; p38α^Fab-KO^ *n =* 5 mice and 5 pictures of each mouse). *p < 0.05, ***p < 0.001 Fab-Cre versus p38α^Fab-KO^ (*t* test or Welch’s test when variances were different). See also S1 Data. BAT, brown adipose tissue; eWAT, epididymal white fat; HFD, high-fat diet; UCP1, uncoupling protein 1.
(TIF)

S6 Fig Specificity of UCP1 antibody, related to Fig 3. Western blot analysis of UCP1 in eWAT from Fab-Cre and p38α^Fab-KO^ mice fed with an HFD. BAT from control mice (diluted 1/10) was used as positive control. Nondiluted BAT and eWAT from UCP1^−/−^ mice were used as negative controls. Each line represents a different mouse. Two different exposures are showed. BAT, brown adipose tissue; eWAT, epididymal white fat; HFD, high-fat diet; UCP1, uncoupling protein 1.
(TIF)

S7 Fig HFD-fed p38α^Fab-KO^ mice have higher iWAT and lower eWAT browning, related to Fig 3. Fab-Cre and p38α^Fab-KO^ mice were fed with HFD for 8 weeks. Immunoblot analysis of UCP1 protein levels and Creb, ATF2, p38, AMPK, and ACC phosphorylation in lysates from iWAT (panel a) or eWAT (panel b). Quantifications are shown in lower panels (mean ± SEM, Fab-Cre *n =* 4–10 mice; p38α^Fab-KO^ *n =* 4–10 mice). *p < 0.05, Fab-Cre versus p38α^Fab-KO^ (*t* test or Welch’s test when variances were different). See also S1 Data. ACC, acetyl-CoA carboxylase; AMPK, 5' adenosine monophosphate-activated protein kinase; ATF2, activating transcription factor 2; Creb, cAMP response element-binding; eWAT, epididymal white fat; HFD, high-fat diet; iWAT, inguinal fat; UCP1, uncoupling protein 1.
(TIF)

S8 Fig p38α controls brown adipocyte differentiation in vitro, related to Fig 3. (a–e) Primary adipocytes isolated from interscapular BAT of Fab-Cre and p38α^Fab-KO^ were differentiated in vitro. (a) Immunoblot analysis of PGC1α and UCP1 protein levels (left panel) and qRT-PCR analysis of browning genes mRNA expression (right panel). mRNA expression was normalized to the amount of *Gapdh* mRNA. (mean ± SEM, Fab-Cre *n =* 6 mice; p38α^Fab-KO^ *n =* 6 wells from 2 independent experiments). qRT-PCR analysis of mRNA expression of adipogenic (panel b), glycolytic (panel b), β-oxidation (panel c), and lipogenic (panel c) genes in in vitro–differentiated primary brown adipocytes. mRNA expression was normalized to the amount of *Gapdh* mRNA (mean ± SEM, a representative experiment is shown; Fab-Cre *n =* 6 wells; p38α^Fab-KO^ *n =* 6 wells). (d) Oil red O staining of primary brown adipocytes after 10 days of differentiation in vitro. (e) Confocal imaging of Fab-Cre and p38α^Fab-KO^ primary brown adipocytes stained with Mitotracker Deep Red (red) and Bodipy (green). Scale bar: 10 μm (left panel). Quantification of cellular triglyceride content in in vitro–differentiated primary brown adipocytes (right panel) (mean ± SEM, a representative experiment is shown; Fab-Cre *n =* 4 wells; p38α^Fab-KO^ *n =* 5 wells). Statistically significant differences between Fab-Cre and p38α^Fab-KO^ brown adipocytes are indicated: *p < 0.05; **p < 0.01; ***p < 0.001 (*t* test or Welch’s test when variances were different). (f, g) Primary adipocytes isolated from subcutaneous white fat of Fab-Cre and p38α^Fab-KO^ were differentiated in vitro. (f) qRT-PCR analysis of mRNA expression of adipogenic genes in in vitro–differentiated primary white adipocytes. mRNA expression was normalized to the amount of *Gapdh* mRNA (mean ± SEM, a representative experiment is shown; Fab-Cre *n =* 9 wells; p38α^Fab-KO^ *n =* 8 wells from 3 independent experiments), *p < 0.05 (Welch’s test). (g) Oil red O staining of primary white adipocytes after 9 days of differentiation in vitro. See also S1 Data. Acaca, acetyl-CoA carboxylase 1; Acox1, acyl-CoA oxidase 1; Adipoq, adiponectin; BAT, brown adipose tissue; Cidea, Cell death activator CIDE-A; Cox7a1, cytochrome C oxidase subunit 7a1; Cpt1a, carnitine palmitoyltransferase 1a; Dgat1, diacylglycerol acyltransferase-1; Fasn; fatty acid synthase; G6pc, glucose-6-phosphatase catalytic subunit; Glys2, glycogen synthase 2; Pepck, phosphoenolpyruvate carboxykinase; PGC1α, proliferator-activated receptor gamma coactivator 1α; Plin1, perilipin 1; Ppard, peroxisome proliferator-activated receptor delta; Pparg, peroxisome proliferator-activated receptor gamma; Ppargc1a, peroxisome proliferator-activated receptor gamma coactivator 1-alpha; Ppargc1b, peroxisome proliferator-activated receptor gamma coactivator 1-beta; Prdm16, PR domain zinc finger protein 16; qRT-PCR quantitative real-time polymerase chain reaction; Scd1, stearoyl-CoA desaturase-1; UCP1, uncoupling protein 1.
(TIF)

S9 Fig Activation of p38 isoforms in Fab-Cre and p38α^Fab-KO^ mice after HFD, related to Fig 3. (a) Phosphorylation of p38 isoforms in adipocytes detected with cell signal antibody #9211. Western blot analysis of the different p38 isoforms activation in adipocytes from WT and p38γ/δ^−/−^ cells. (b) Immunoblot analysis of p38 phosphorylation in BAT, eWAT, iWAT, sWAT, and pWAT lysates from ND-fed Fab-Cre and p38α^Fab-KO^ mice. (c) Effect of SB203580 on phosphorylation of p38 isoforms. Western blot analysis of phospho p38 in brown preadipocytes from Fab-Cre mice treated with DMSO, sorbitol (0.5 M, 15 minutes), or sorbitol with SB203580 (10 μM, 1 hour pre treatment) or from p38α^Fab-KO^ mice with DMSO. (d) qRT-PCR analysis of different isoforms of p38 mRNA expression (p38α [*Mapk14*], p38β [*Mapk11*], p38γ [*Mapk12*], p38δ [*Mapk13*]) in BAT and eWAT from control mice (Fab-Cre) after an ND or an HFD for 8 weeks. mRNA expression was normalized to the amount of *Gapdh* mRNA (mean ± SEM, ND *n =* 6–9 mice; HFD *n =* 14 mice). (e) Comparison of p38 isoforms mRNA expression by qRT-PCR analysis in BAT from ND-fed Fab-Cre and p38α^Fab-KO^ mice. mRNA expression was normalized to the amount of *Gapdh* mRNA (mean ± SEM, Fab-Cre *n =* 6 mice; p38α^Fab-KO^ *n =* 7 mice). (f) Comparison of p38 isoforms mRNA expression by qRT-PCR analysis in eWAT from ND- and HFD-fed Fab-Cre and p38α^Fab-KO^ mice. mRNA expression was normalized to the amount of *Gapdh* mRNA (mean ± SEM, Fab-Cre *n =* 7–14 mice; p38α^Fab-KO^ *n =* 7–9 mice). *p < 0.05; **p < 0.01; ***p < 0.001; Fab-Cre versus p38δ^Fab-KO^ (*t* test or Welch’s test when variances were different). See also S1 Data. BAT, brown adipose tissue; eWAT, epididymal white fat; GTT, glucose tolerance test; HFD, high-fat diet; iWAT, inguinal fat; ND, normal-chow diet; pWAT, perirenal WAT; qRT-PCR, quantitative real-time polymerase chain reaction; sWAT, subcutaneous fat; WT, wild type.
(TIF)

S10 Fig Brown fat from p38δ^Fab-KO^ mice presents a decrease in BAT activity, related to Fig 6. Fab-Cre and p38δ^Fab-KO^ mice were fed with an ND for 8 weeks. (a) Weight of eWAT, sWAT, iWAT, pWAT, BAT, and liver with respect to tibia length in ND-fed Fab-Cre and p38δ^Fab-KO^ mice (mean ± SEM, Fab-Cre *n =* 6 mice; p38δ^Fab-KO^ *n =* 6 mice). (b) qRT-PCR analysis of mRNA expression of browning genes in BAT isolated from ND-fed Fab-Cre and p38δ^Fab-KO^ mice. mRNA expression was normalized to the amount of *Gapdh* mRNA. (c) Western blot analysis of PKA activation in BAT from Fab-Cre and p38δ^Fab-KO^. Each line represents a different mouse (*n =* 6) (mean ± SEM, Fab-Cre *n =* 6 mice; p38δ^Fab-KO^ *n =* 6 mice). *p < 0.05; **p < 0.01; Fab-Cre versus p38δ^Fab-KO^ (*t* test or Welch’s test when variances were different). See also S1 Data. BAT, brown adipose tissue; Cidea, Cell death activator CIDE-A; eWAT, epididymal white fat; GTT, glucose tolerance test; iWAT, inguinal fat; ND, normal-chow diet; pWAT, perirenal WAT; PKA, protein kinase A; Ppargc1a, peroxisome proliferator-activated receptor gamma coactivator 1-alpha; Prdm16, PR domain zinc finger protein 16; qRT-PCR quantitative real-time polymerase chain reaction; sWAT, subcutaneous fat; UCP1, uncoupling protein 1.
(TIF)

S11 Fig p38δ^Fab-KO^ mice have higher body weight and lower temperature when fed an HFD, related to Fig 6. Fab-Cre and p38δ^Fab-KO^ mice were fed with an HFD for 8 weeks. (a) Body weight at the end of the treatment (mean ± SEM, Fab-Cre *n =* 8 mice; p38δ^Fab-KO^ *n =* 7 mice). (b) NMR analysis of body mass and fat mass in p38δ^Fab-KO^ and Fab-Cre mice after 8 weeks of HFD (mean ± SEM, Fab-Cre *n =* 8 mice; p38δ^Fab-KO^ *n =* 7 mice). (c) Weight of eWAT, sWAT, iWAT, pWAT, BAT, and liver with respect to tibia length (mean ± SEM, Fab-Cre *n =* 8 mice; p38δ^Fab-KO^ *n =* 7 mice). (d) Skin temperature surrounding interscapular BAT in HFD-fed Fab-Cre and p38δ^Fab-KO^. Right panels show representative infrared thermal images (mean ± SEM, Fab-Cre *n =* 8 mice; p38δ^Fab-KO^ *n =* 7 mice). (e) qRT-PCR analysis of mRNA expression of browning genes in BAT isolated from HFD-fed Fab-Cre and p38δ^Fab-KO^ mice. mRNA expression was normalized to the amount of *Gapdh* mRNA (mean ± SEM, Fab-Cre *n =* 5 mice; p38δ^Fab-KO^ *n =* 6 mice). (f) Immunoblot of UCP1 protein levels in p38δ^Fab-KO^ and Fab-Cre mice after 8 weeks of HFD. Quantification is shown on the right panel (mean ± SEM, Fab-Cre *n =* 5 mice; p38δ^Fab-KO^ *n =* 6 mice). *p < 0.05; **p < 0.01; ***p < 0.001; Fab-Cre versus p38δ^Fab-KO^ (*t* test or Welch’s test when variances were different). See also S1 Data. BAT, brown adipose tissue; Cidea, Cell death activator CIDE-A; eWAT, epididymal white fat; GTT, glucose tolerance test; HFD, high-fat diet; IR temperature, infrared temperature; iWAT, inguinal fat; Ppargc1a, peroxisome proliferator-activated receptor gamma coactivator 1-alpha; Ppargc1b, peroxisome proliferator-activated receptor gamma coactivator 1-beta; Prdm16, PR domain zinc finger protein 16; pWAT, perirenal WAT; qRT-PCR quantitative real-time polymerase chain reaction; sWAT, subcutaneous fat; UCP1, uncoupling protein 1.
(TIF)

S1 Table Characteristics of patients and controls for human visceral fat samples. (DOCX)

S2 Table Characteristics of patients and controls for human sWAT samples. sWAT, subcutaneous fat.
(DOCX)

S3 Table Primers used for gene amplification. (DOCX)

S1 Data Numerical data used in figures. (XLSX)

S1 Text Figure legend from S1 Fig. (DOCX)

S2 Text Figure legend from S2 Fig. (DOCX)

S3 Text Figure legend from S3 Fig. (DOCX)

S4 Text Figure legend from S4 Fig. (DOCX)

S5 Text Figure legend from S5 Fig. (DOCX)

S6 Text Figure legend from S6 Fig. (DOCX)

S7 Text Figure legend from S7 Fig. (DOCX)

S8 Text Figure legend from S8 Fig. (DOCX)

S9 Text Figure legend from S9 Fig. (DOCX)

S10 Text Figure legend from S10 Fig. (DOCX)

S11 Text Figure legend from S11 Fig. (DOCX)

## Abbreviations

AMPK: 5' adenosine monophosphate-activated protein kinase
ALT: alanine aminotransferase
AST: aspartate aminotransferase
ATF2: activating transcription factor 2
BAT: brown adipose tissue
BM: bone marrow
BMI: body mass index
BrdU: bromodeoxyuridine
Creb: cAMP response element-binding
eWAT: epididymal fat
FACS: fluorescence-assisted cell sorting
FCCP: carbonyl cyanide-4-(trifluoromethoxy)phenylhydrazone
GLUT4: glucose transporter type 4
GTT: glucose tolerance test
HFD: high-fat diet
IR temperature: infrared temperature
ISO: isoproterenol
ITT: insulin tolerance test
iWAT: inguinal fat
ND: normal-chow diet
NE: norepinephrine
NMR: nuclear magnetic resonance
OCR: oxygen consumption rate
PGC1α: proliferator-activated receptor gamma coactivator 1α
PKA: protein kinase A
pWAT: perirenal fat
qRT-PCR: quantitative real-time polymerase chain reaction
sWAT: subcutaneous fat
UCP1: uncoupling protein 1
WAT: white adipose tissue
WT: wild-type
