# Supplementary figures and images for: p38α blocks brown adipose tissue thermogenesis through p38δ inhibition
Source: PLoS Biol. 2018 Jul 6;16(7):e2004455. doi: 10.1371/journal.pbio.2004455 (PMC6051667; doi:10.1371/journal.pbio.2004455)

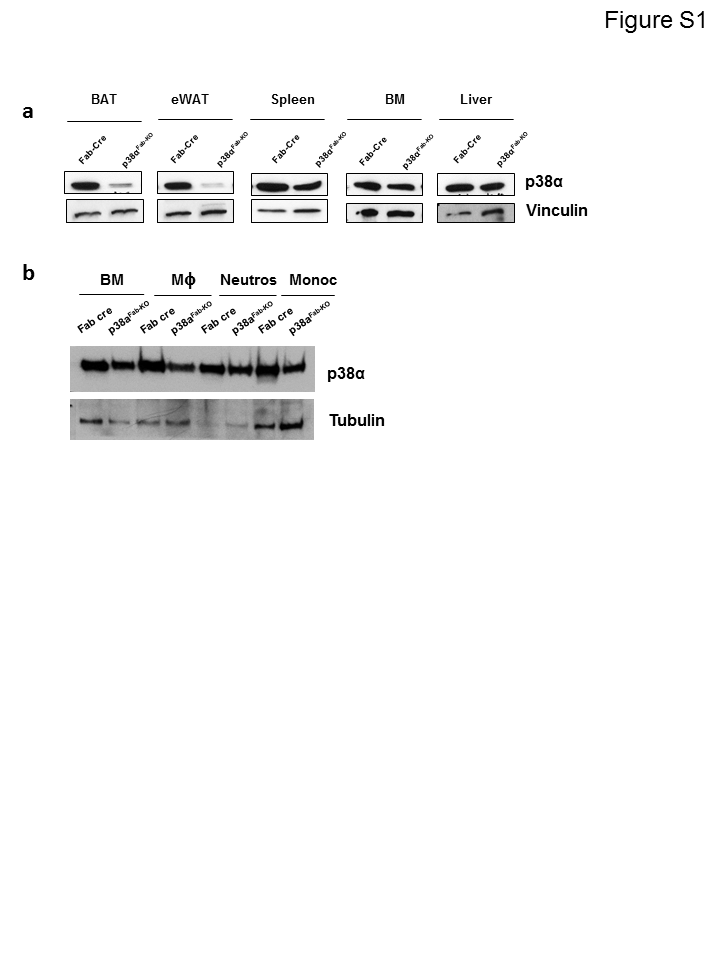

Supplement: S1 Fig — (a) Western blot analysis of p38α expression in BAT, eWAT, spleen, BM, and liver isolated from p38αFab-KO and control (Fab-Cre) mice. (b) Western blot analysis of p38α expression in BM, Mɸ, Neutros, and Mono. Mɸ and Neutros were sorted from spleen, and Mono from BM by FACS. BAT, brown adipose tissue; BM, bone marrow; eWAT, epididymal white adipose tissue; FACS, fluorescence assisted-cell sorting; Mɸ, macrophages; Mono, monocytes; Neutros, neutrophils. (TIF) [file pbio.2004455.s001.tif]

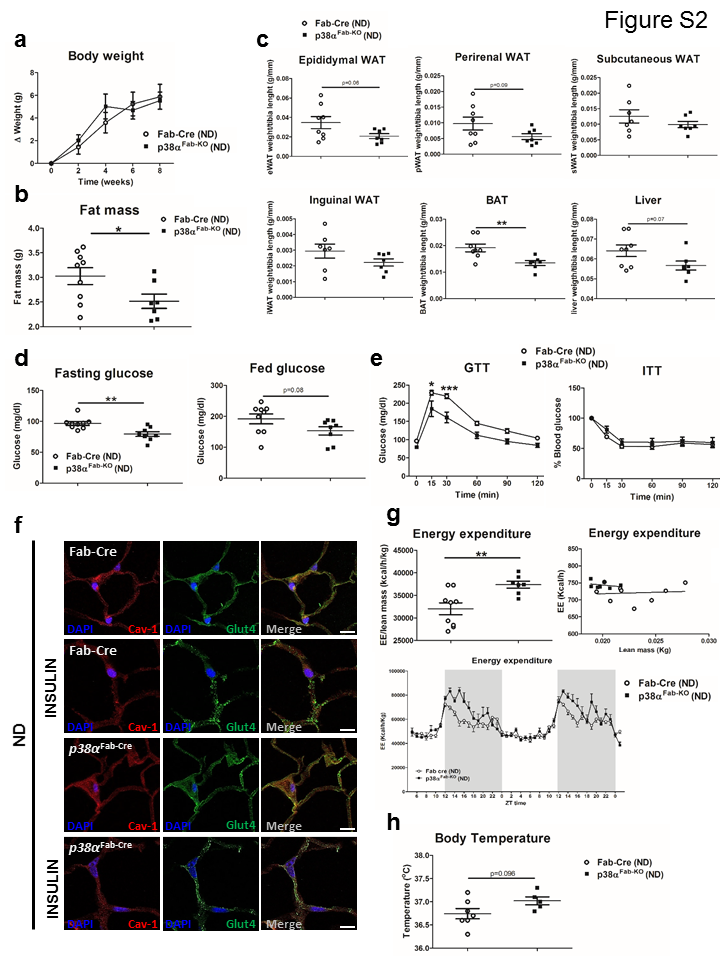

Supplement: S2 Fig — (a) Body weight time course in Fab-Cre and p38αFab-KO male (8- to 10-week-old) mice fed an ND over 8 weeks. Data are presented as the increase above initial weight (mean ± SEM, Fab-Cre n = 9 mice; p38αFab-KO n = 8 mice). (b) NMR analysis of fat mass in p38αFab-KO and Fab-Cre mice after 8 weeks of ND (mean ± SEM, Fab-Cre n = 9 mice; p38αFab-KO n = 7 mice). (c) Weight of eWAT, pWAT, sWAT, iWAT, BAT, and liver relativized to tibia length (mean ± SEM, Fab-Cre n = 8 mice; p38αFab-KO n = 7 mice). (d) Fasting and fed blood glucose in Fab-Cre and p38αFab-KO mice fed an ND (8 weeks) (mean ± SEM, Fab-Cre n = 9 mice; p38αFab-KO n = 8 mice). (e) GTT and ITT in Fab-Cre and p38αFab-KO mice fed HFD for 8 weeks. Mice were fasted overnight (for GTT) or 1 hour (for ITT), and blood glucose concentration was measured in mice given intraperitoneal injections of glucose (1 g/kg of total body weight) or insulin (0.75 U/kg of total body weight). (mean ± SEM, Fab-Cre n = 9 mice; p38αFab-KO n = 8 mice). (f) Immunohistochemistry of eWAT sections using anti-GLUT4 (green), anti-Cav-1 (red) antibodies, and the nuclear dye DAPI (blue). Location of GLUT4 was analysed in mice treated with or without insulin (1.5 IU/kg) for 15 minutes after overnight fasting. Scale bar: 20 μm. (g) Comparison of energy balance between ND-fed Fab-Cre and p38αFab-KO mice. ND-fed mice were examined in a metabolic cage over a 3-day period to measure EE. EE levels corrected by lean mass (left panel), expressed as ANCOVA analysis (right panel) and hour by hour over a 48-hour period (lower panel) are shown (mean ± SEM, Fab-Cre n = 9 mice; p38αFab-KO n = 7 mice). (h) Body temperature of ND-fed Fab-Cre and p38αFab-KO mice (mean ± SEM, Fab-Cre n = 7 mice; p38αFab-KO n = 5 mice). *p < 0.05; **p < 0.01; ***p < 0.001 Fab-Cre versus p38αFab-KO (2-way ANOVA coupled with Bonferroni’s post-tests or t test or Welch’s test when variances were different). See also S1 Data. BAT, brown adipose tissue; Cav-1, caveolin-1; EE, energy expendi [file pbio.2004455.s002.tif]

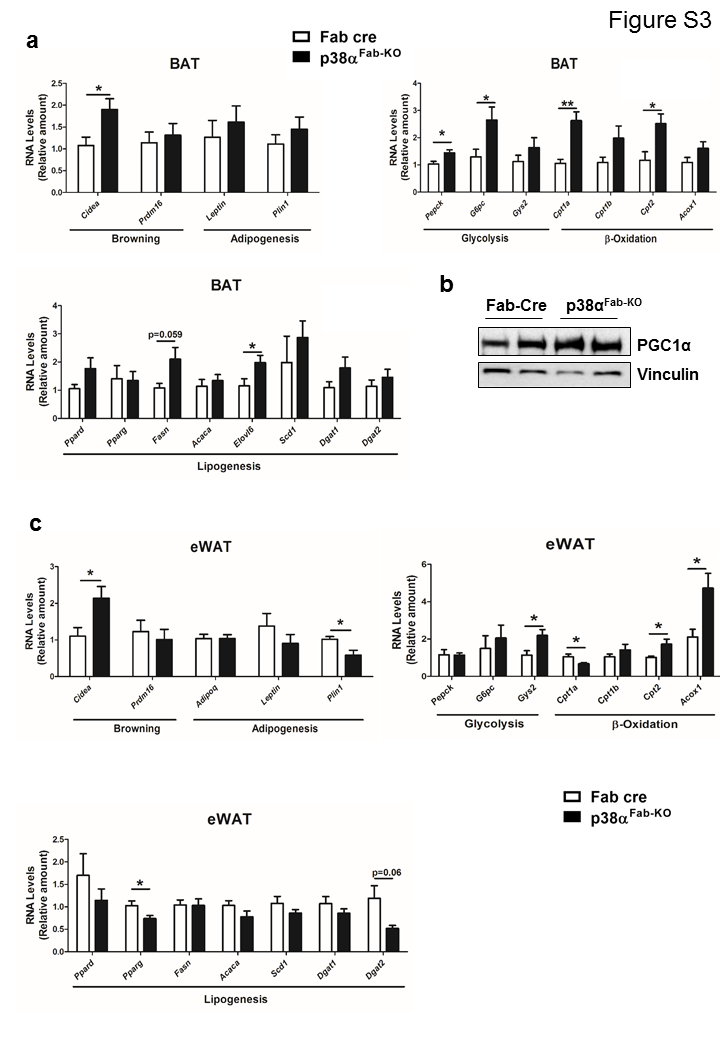

Supplement: S3 Fig — (a) qRT-PCR analysis of mRNA expression of browning, adipogenic, glycolytic, β-oxidation and lipogenic genes from BAT of ND-fed Fab-Cre and p38αFab-KO mice. mRNA expression was normalized to the amount of Gapdh mRNA. (b) Immunoblot analysis of PGC1α protein levels in BAT of ND-fed Fab-Cre and p38αFab-KO mice (c) qRT-PCR analysis of mRNA expression of browning, adipogenic, glycolytic, β-oxidation and lipogenic genes from eWAT of ND-fed Fab-Cre and p38αFab-KO mice. mRNA expression was normalized to the amount of Gapdh mRNA (mean ± SEM, Fab-Cre n = 7 mice; p38αFab-KO n = 7 mice). *p < 0.05; **p < 0.01. Fab-Cre versus p38αFab-KO (t test or Welch’s test when variances were different). See also S1 Data. Acaca, acetyl-CoA carboxylase 1; Acox1, acyl-CoA oxidase 1; Adipoq, Adiponectin; BAT, brown adipose tissue; cidea, cell death activator; Cpt1a, carnitine palmitoyltransferase 1A; Cpt2, carnitine palmitoyltransferase 2; Dgat1, diacylglycerol acyltransferase-1; Dgat2, diacylglycerol acyltransferase-2; Elovl, fatty acid elongase 6; eWAT, epididymal fat; Fasn, fatty acid synthase; G6pc, glucose-6-phosphatase catalytic subunit; Glys2, glycogen synthase 2; ND, normal-chow diet; Pepck, phosphoenolpyruvate carboxykinase; PGC1α, proliferator-activated receptor gamma coactivator 1α; Plin1, perilipin 1; Ppard, peroxisome proliferator-activated receptor delta; Pparg, peroxisome proliferator-activated receptor gamma; Prdm16, PR domain zinc finger protein 16; qRT-PCR, quantitative real-time polymerase chain reaction; Scd1, stearoyl-CoA desaturase-1. (TIF) [file pbio.2004455.s003.tif]

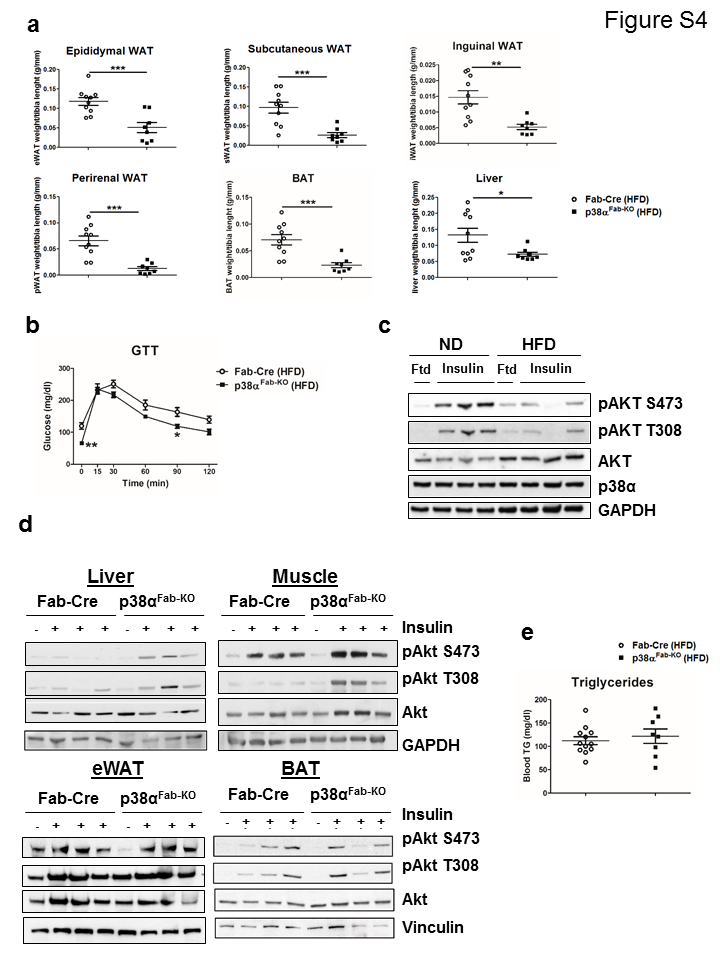

Supplement: S4 Fig — Fab-Cre and p38αFab-KO mice were fed an HFD for 8 weeks. (a) Weight of eWAT, sWAT, iWAT, pWAT, BAT, and liver relativized to tibia length (mean ± SEM, Fab-Cre n = 10 mice; p38αFab-KO n = 8 mice). (b) GTT in Fab-Cre and p38αFab-KO mice fed the HFD for 8 weeks. Mice were fasted overnight, and blood glucose concentration was measured in mice given intraperitoneal injections of glucose (1 g/kg of lean mass) (mean ± SEM, Fab-Cre n = 5 mice; p38αFab-KO n = 6 mice). (c) Western blot analysis of Akt activation in the liver from Fab-Cre mice fed with ND or HFD. Mice were treated without or with insulin (1.5 IU/kg) for 15 minutes after overnight fasting. Each line represents a different mouse. (d) Western blot analysis of Akt activation in the liver, skeletal muscle, eWAT, and BAT from mice fed with HFD. Mice were treated without or with insulin (1.5 IU/kg) for 15 minutes after overnight fasting. Each line represents a different mouse. (e) Triglyceride content in blood samples from Fab-Cre and p38αFab-KO mice (mean ± SEM, Fab-Cre n = 12 mice; p38αFab-KO n = 8 mice). *p < 0.05, ***p < 0.001 Fab-Cre versus p38αFab-KO (2-way ANOVA coupled with Bonferroni’s post-tests or t test or Welch’s test when variances were different). See also S1 Data. BAT, brown adipose tissue; eWAT, epididymal white fat; GTT, glucose tolerance test; Fts, Fasted; HFD, high-fat diet; iWAT, inguinal fat; ND, normal-chow diet; pWAT, perirenal WAT; sWAT, subcutaneous fat. (TIF) [file pbio.2004455.s004.tif]

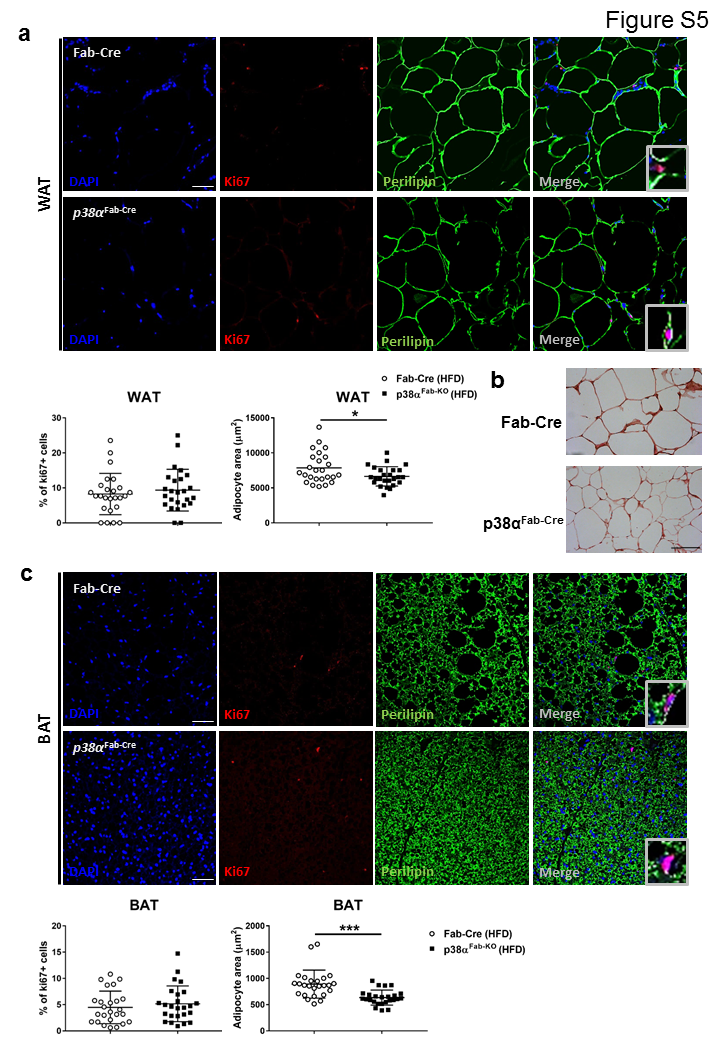

Supplement: S5 Fig — Fab-Cre and p38αFab-KO mice were fed an HFD for 8 weeks. (a) Immunohistochemistry of eWAT sections using anti-Ki67 (red), anti-perilipin (green) antibodies, and the nuclear dye DAPI (blue) (upper panel). Scale bar: 20 μm. A positive cell is shown in a bigger magnification for each genotype. Quantification of proliferation and adipocyte size are shown (lower panel) (mean ± SEM, Fab-Cre n = 5 mice; p38αFab-KO n = 5 mice and 5 pictures of each mouse). (b) Staining of UCP1 after 8 weeks of HFD in eWAT. Representative pictures are shown from Fab-Cre n = 6 mice; p38αFab-KO n = 6 mice with 3 pictures of each mouse. Scale bar: 50 μm. (c) Immunohistochemistry of BAT sections using anti-Ki67 (red), anti-perilipin (green) antibodies, and the nuclear dye DAPI (blue) (upper panel). Scale bar: 20 μm. A positive cell is shown in a bigger magnification for each genotype. Quantification of proliferation and adipocyte size are shown (lower panel) (mean ± SEM, Fab-Cre n = 5 mice; p38αFab-KO n = 5 mice and 5 pictures of each mouse). *p < 0.05, ***p < 0.001 Fab-Cre versus p38αFab-KO (t test or Welch’s test when variances were different). See also S1 Data. BAT, brown adipose tissue; eWAT, epididymal white fat; HFD, high-fat diet; UCP1, uncoupling protein 1. (TIF) [file pbio.2004455.s005.tif]

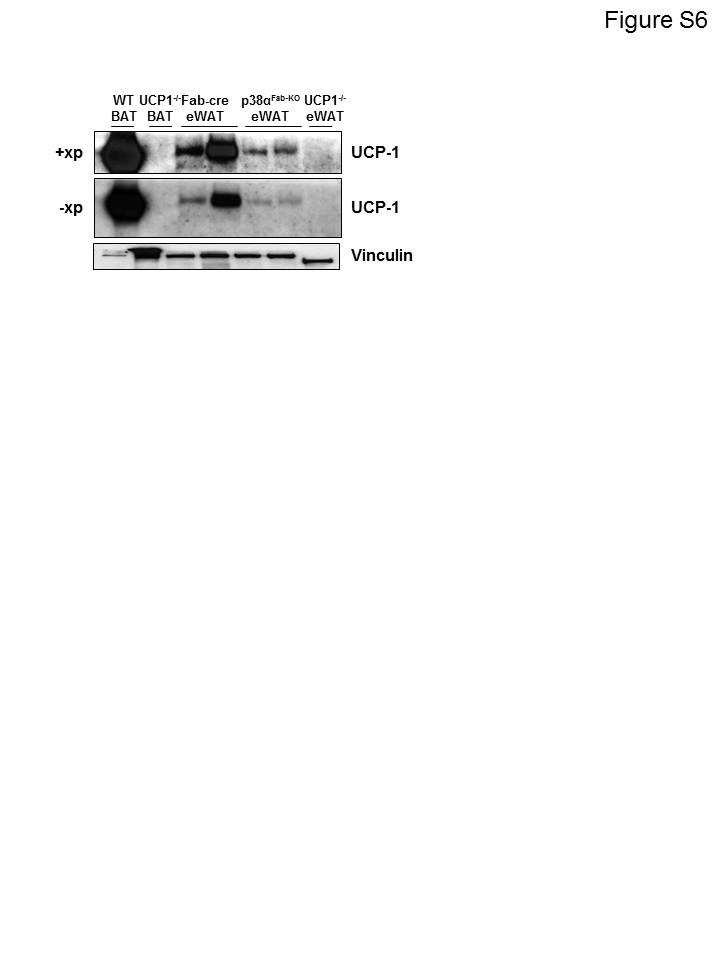

Supplement: S6 Fig — Western blot analysis of UCP1 in eWAT from Fab-Cre and p38αFab-KO mice fed with an HFD. BAT from control mice (diluted 1/10) was used as positive control. Nondiluted BAT and eWAT from UCP1−/− mice were used as negative controls. Each line represents a different mouse. Two different exposures are showed. BAT, brown adipose tissue; eWAT, epididymal white fat; HFD, high-fat diet; UCP1, uncoupling protein 1. (TIF) [file pbio.2004455.s006.tif]

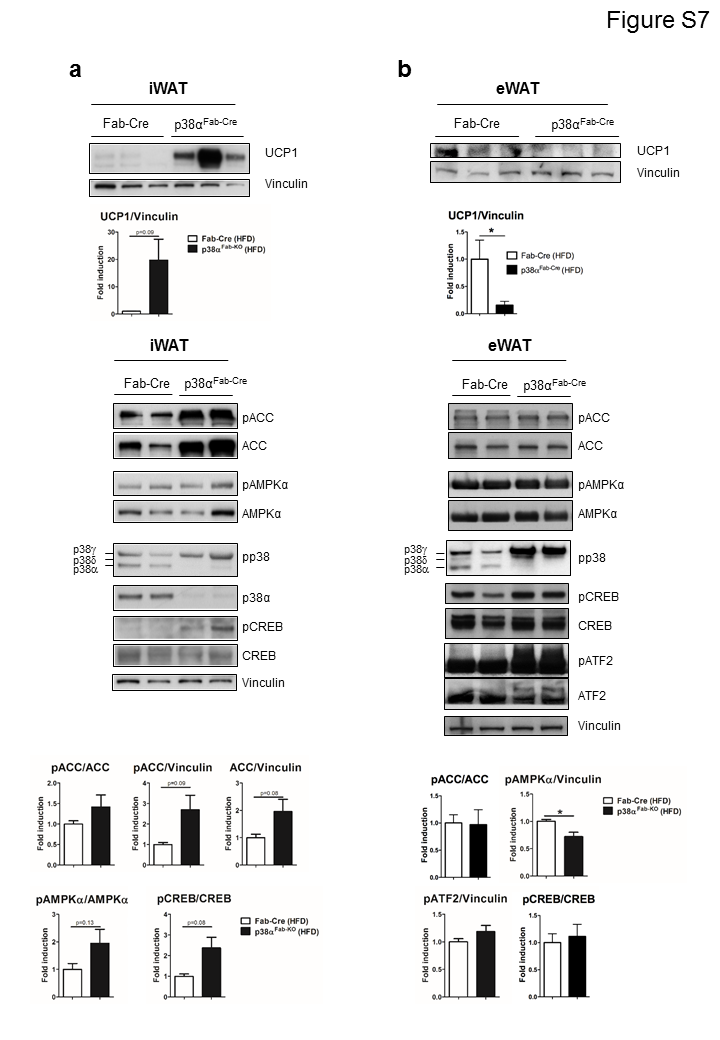

Supplement: S7 Fig — Fab-Cre and p38αFab-KO mice were fed with HFD for 8 weeks. Immunoblot analysis of UCP1 protein levels and Creb, ATF2, p38, AMPK, and ACC phosphorylation in lysates from iWAT (panel a) or eWAT (panel b). Quantifications are shown in lower panels (mean ± SEM, Fab-Cre n = 4–10 mice; p38αFab-KO n = 4–10 mice). *p < 0.05, Fab-Cre versus p38αFab-KO (t test or Welch’s test when variances were different). See also S1 Data. ACC, acetyl-CoA carboxylase; AMPK, 5' adenosine monophosphate-activated protein kinase; ATF2, activating transcription factor 2; Creb, cAMP response element-binding; eWAT, epididymal white fat; HFD, high-fat diet; iWAT, inguinal fat; UCP1, uncoupling protein 1. (TIF) [file pbio.2004455.s007.tif]

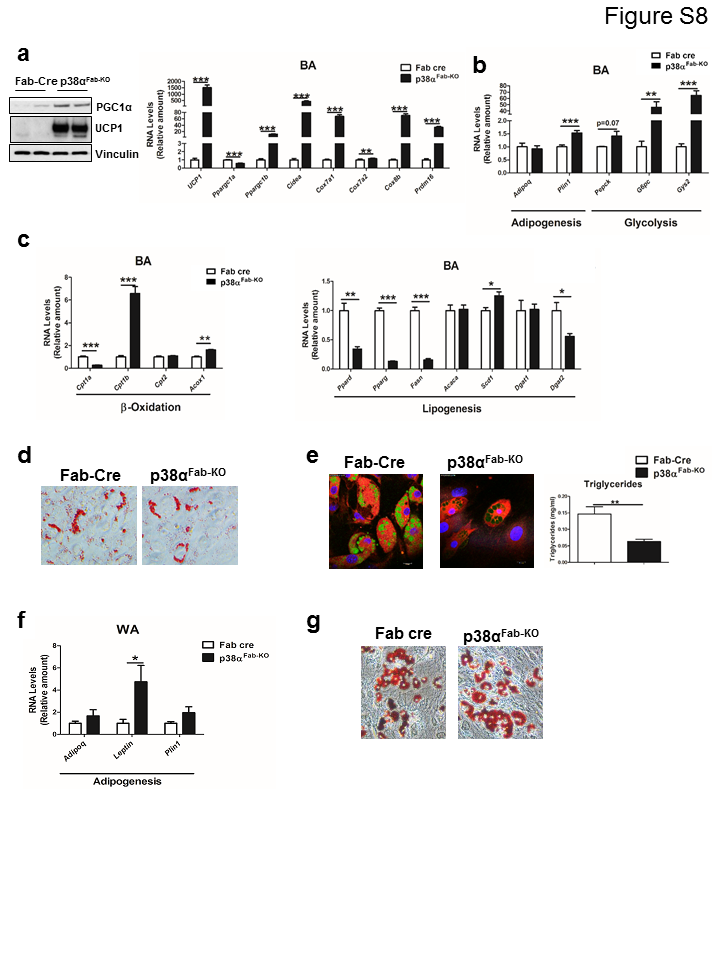

Supplement: S8 Fig — (a–e) Primary adipocytes isolated from interscapular BAT of Fab-Cre and p38αFab-KO were differentiated in vitro. (a) Immunoblot analysis of PGC1α and UCP1 protein levels (left panel) and qRT-PCR analysis of browning genes mRNA expression (right panel). mRNA expression was normalized to the amount of Gapdh mRNA. (mean ± SEM, Fab-Cre n = 6 mice; p38αFab-KO n = 6 wells from 2 independent experiments). qRT-PCR analysis of mRNA expression of adipogenic (panel b), glycolytic (panel b), β-oxidation (panel c), and lipogenic (panel c) genes in in vitro–differentiated primary brown adipocytes. mRNA expression was normalized to the amount of Gapdh mRNA (mean ± SEM, a representative experiment is shown; Fab-Cre n = 6 wells; p38αFab-KO n = 6 wells). (d) Oil red O staining of primary brown adipocytes after 10 days of differentiation in vitro. (e) Confocal imaging of Fab-Cre and p38αFab-KO primary brown adipocytes stained with Mitotracker Deep Red (red) and Bodipy (green). Scale bar: 10 μm (left panel). Quantification of cellular triglyceride content in in vitro–differentiated primary brown adipocytes (right panel) (mean ± SEM, a representative experiment is shown; Fab-Cre n = 4 wells; p38αFab-KO n = 5 wells). Statistically significant differences between Fab-Cre and p38αFab-KO brown adipocytes are indicated: *p < 0.05; **p < 0.01; ***p < 0.001 (t test or Welch’s test when variances were different). (f, g) Primary adipocytes isolated from subcutaneous white fat of Fab-Cre and p38αFab-KO were differentiated in vitro. (f) qRT-PCR analysis of mRNA expression of adipogenic genes in in vitro–differentiated primary white adipocytes. mRNA expression was normalized to the amount of Gapdh mRNA (mean ± SEM, a representative experiment is shown; Fab-Cre n = 9 wells; p38αFab-KO n = 8 wells from 3 independent experiments), *p < 0.05 (Welch’s test). (g) Oil red O staining of primary white adipocytes after 9 days of differentiation in vitro. See also S1 Data. Acaca, acetyl-CoA carboxylase 1; Aco [file pbio.2004455.s008.tif]

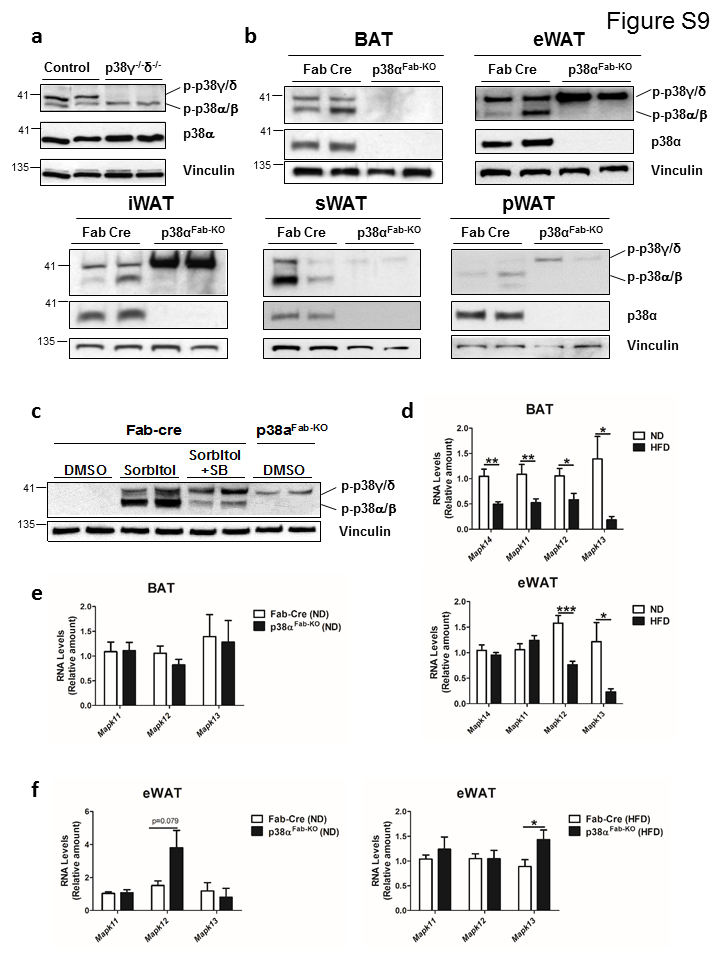

Supplement: S9 Fig — (a) Phosphorylation of p38 isoforms in adipocytes detected with cell signal antibody #9211. Western blot analysis of the different p38 isoforms activation in adipocytes from WT and p38γ/δ−/− cells. (b) Immunoblot analysis of p38 phosphorylation in BAT, eWAT, iWAT, sWAT, and pWAT lysates from ND-fed Fab-Cre and p38αFab-KO mice. (c) Effect of SB203580 on phosphorylation of p38 isoforms. Western blot analysis of phospho p38 in brown preadipocytes from Fab-Cre mice treated with DMSO, sorbitol (0.5 M, 15 minutes), or sorbitol with SB203580 (10 μM, 1 hour pre treatment) or from p38αFab-KO mice with DMSO. (d) qRT-PCR analysis of different isoforms of p38 mRNA expression (p38α [Mapk14], p38β [Mapk11], p38γ [Mapk12], p38δ [Mapk13]) in BAT and eWAT from control mice (Fab-Cre) after an ND or an HFD for 8 weeks. mRNA expression was normalized to the amount of Gapdh mRNA (mean ± SEM, ND n = 6–9 mice; HFD n = 14 mice). (e) Comparison of p38 isoforms mRNA expression by qRT-PCR analysis in BAT from ND-fed Fab-Cre and p38αFab-KO mice. mRNA expression was normalized to the amount of Gapdh mRNA (mean ± SEM, Fab-Cre n = 6 mice; p38αFab-KO n = 7 mice). (f) Comparison of p38 isoforms mRNA expression by qRT-PCR analysis in eWAT from ND- and HFD-fed Fab-Cre and p38αFab-KO mice. mRNA expression was normalized to the amount of Gapdh mRNA (mean ± SEM, Fab-Cre n = 7–14 mice; p38αFab-KO n = 7–9 mice). *p < 0.05; **p < 0.01; ***p < 0.001; Fab-Cre versus p38δFab-KO (t test or Welch’s test when variances were different). See also S1 Data. BAT, brown adipose tissue; eWAT, epididymal white fat; GTT, glucose tolerance test; HFD, high-fat diet; iWAT, inguinal fat; ND, normal-chow diet; pWAT, perirenal WAT; qRT-PCR, quantitative real-time polymerase chain reaction; sWAT, subcutaneous fat; WT, wild type. (TIF) [file pbio.2004455.s009.tif]

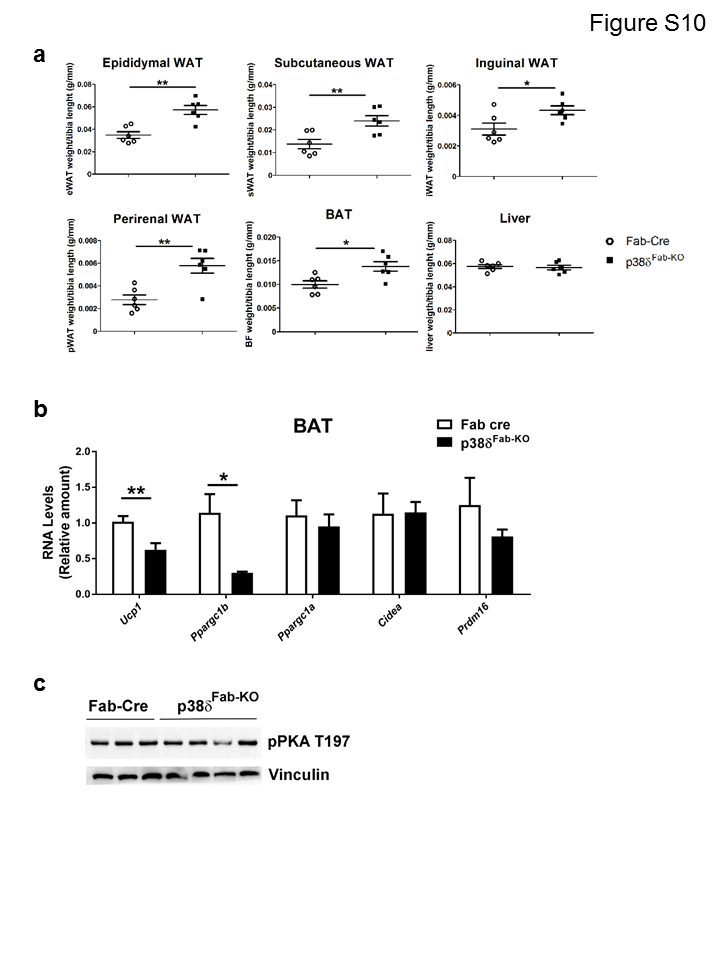

Supplement: S10 Fig — Fab-Cre and p38δFab-KO mice were fed with an ND for 8 weeks. (a) Weight of eWAT, sWAT, iWAT, pWAT, BAT, and liver with respect to tibia length in ND-fed Fab-Cre and p38δFab-KO mice (mean ± SEM, Fab-Cre n = 6 mice; p38δFab-KO n = 6 mice). (b) qRT-PCR analysis of mRNA expression of browning genes in BAT isolated from ND-fed Fab-Cre and p38δFab-KO mice. mRNA expression was normalized to the amount of Gapdh mRNA. (c) Western blot analysis of PKA activation in BAT from Fab-Cre and p38δFab-KO. Each line represents a different mouse (n = 6) (mean ± SEM, Fab-Cre n = 6 mice; p38δFab-KO n = 6 mice). *p < 0.05; **p < 0.01; Fab-Cre versus p38δFab-KO (t test or Welch’s test when variances were different). See also S1 Data. BAT, brown adipose tissue; Cidea, Cell death activator CIDE-A; eWAT, epididymal white fat; GTT, glucose tolerance test; iWAT, inguinal fat; ND, normal-chow diet; pWAT, perirenal WAT; PKA, protein kinase A; Ppargc1a, peroxisome proliferator-activated receptor gamma coactivator 1-alpha; Prdm16, PR domain zinc finger protein 16; qRT-PCR quantitative real-time polymerase chain reaction; sWAT, subcutaneous fat; UCP1, uncoupling protein 1. (TIF) [file pbio.2004455.s010.tif]

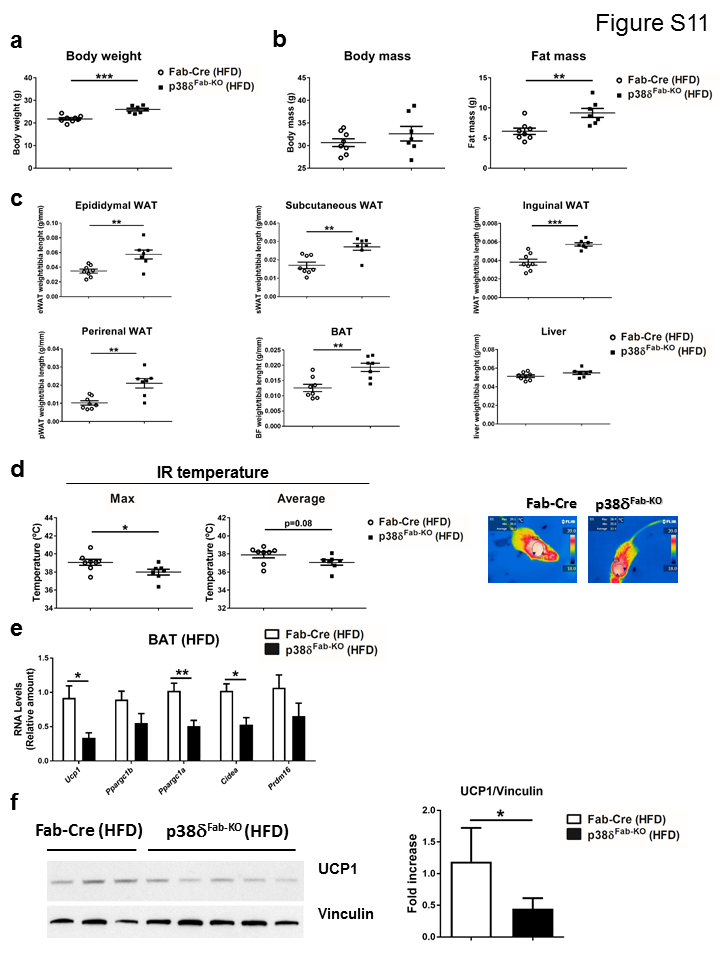

Supplement: S11 Fig — Fab-Cre and p38δFab-KO mice were fed with an HFD for 8 weeks. (a) Body weight at the end of the treatment (mean ± SEM, Fab-Cre n = 8 mice; p38δFab-KO n = 7 mice). (b) NMR analysis of body mass and fat mass in p38δFab-KO and Fab-Cre mice after 8 weeks of HFD (mean ± SEM, Fab-Cre n = 8 mice; p38δFab-KO n = 7 mice). (c) Weight of eWAT, sWAT, iWAT, pWAT, BAT, and liver with respect to tibia length (mean ± SEM, Fab-Cre n = 8 mice; p38δFab-KO n = 7 mice). (d) Skin temperature surrounding interscapular BAT in HFD-fed Fab-Cre and p38δFab-KO. Right panels show representative infrared thermal images (mean ± SEM, Fab-Cre n = 8 mice; p38δFab-KO n = 7 mice). (e) qRT-PCR analysis of mRNA expression of browning genes in BAT isolated from HFD-fed Fab-Cre and p38δFab-KO mice. mRNA expression was normalized to the amount of Gapdh mRNA (mean ± SEM, Fab-Cre n = 5 mice; p38δFab-KO n = 6 mice). (f) Immunoblot of UCP1 protein levels in p38δFab-KO and Fab-Cre mice after 8 weeks of HFD. Quantification is shown on the right panel (mean ± SEM, Fab-Cre n = 5 mice; p38δFab-KO n = 6 mice). *p < 0.05; **p < 0.01; ***p < 0.001; Fab-Cre versus p38δFab-KO (t test or Welch’s test when variances were different). See also S1 Data. BAT, brown adipose tissue; Cidea, Cell death activator CIDE-A; eWAT, epididymal white fat; GTT, glucose tolerance test; HFD, high-fat diet; IR temperature, infrared temperature; iWAT, inguinal fat; Ppargc1a, peroxisome proliferator-activated receptor gamma coactivator 1-alpha; Ppargc1b, peroxisome proliferator-activated receptor gamma coactivator 1-beta; Prdm16, PR domain zinc finger protein 16; pWAT, perirenal WAT; qRT-PCR quantitative real-time polymerase chain reaction; sWAT, subcutaneous fat; UCP1, uncoupling protein 1. (TIF) [file pbio.2004455.s011.tif]
