# Supplementary material for: p38α blocks brown adipose tissue thermogenesis through p38δ inhibition
Source: PLoS Biol. 2018 Jul 6;16(7):e2004455. doi: 10.1371/journal.pbio.2004455 (PMC6051667; doi:10.1371/journal.pbio.2004455)
Supplement: S1 Table — (DOCX) [file pbio.2004455.s012.docx]

**Table S1. Characteristics of patients and controls for human visceral fat samples.**

| Variable | Obese patients  (n = 53) | Controls  (n = 18) | p |
| --- | --- | --- | --- |
| Age (years) | 46.91 (12.37) | 53.22 (15.85) | 0.177 |
| Female:male ratio | 37:16 | 10:8 | 0.269 |
| Hypertension (n) | 26 (49.10) | 6 (33.30) | 0.322 |
| Diabetes mellitus (n) | 14 (26.40) | 2 (11.10) | 0.369 |
| BMI (kg/m^2^) | 48.47 (6.87) | 25.43 (3.47) | <0.0001 |
| Fasting blood sugar (mg/dL) | 113.34 (43.95) | 94.06 (12.81) | 0.050 |
| AST (IU/L) | 23.1 (12.43) | 29.82 (27.03) | 0.280 |
| ALT (IU/L) | 30.5 (17.92) | 70.06 (162.67) | 0.791 |
| Alkaline phosphatase | 75.08 (27.17) | 91.75 (35.63) | 0.072 |
| Bilirubin (mg/dL) | 0.42 (0.13) | 0.63 (0.32) | 0.011 |
| Albumin (mg/dL) | 4.27 (0.25) | 4.45 (0.45) | 0.010 |
| Total cholesterol (mg/dL) | 188.96 (32.78) | 202.69 (41.96) | 0.162 |
| Triglycerides (mg/dL) | 145.33 (71.62) | 136.7 (49.78) | 0.931 |
| LDL-cholesterol (mg/dL) | 111.37 (33.03) | 124.8 (38.69) | 0.276 |
| HDL-cholesterol (mg/dL) | 46.61 (12.57) | 50.63 (18.83) | 0.863 |

Variables are presented as mean (standard deviation) or absolute frequency (percentage) and are compared by means of Mann-Whitney U test or χ^2^ test. BMI: body mass index. AST: aspartate aminotransferase. ALT: alanine aminotransferase.
