# Supplementary material for: p38α blocks brown adipose tissue thermogenesis through p38δ inhibition
Source: PLoS Biol. 2018 Jul 6;16(7):e2004455. doi: 10.1371/journal.pbio.2004455 (PMC6051667; doi:10.1371/journal.pbio.2004455)
Supplement: S2 Table — sWAT, subcutaneous fat. (DOCX) [file pbio.2004455.s013.docx]

**Table S2. Characteristics of patients and controls for human subcutaneous fat samples.**

| Variable | Obese patients  (n = 140) | Controls  (n = 30) | p |
| --- | --- | --- | --- |
| Age (years) | 46,58 (10,96) | 51,7 (18,11) | 0.145 |
| Female:male ratio | 103:37 | 18:12 | 0.136 |
| Hypertension (n) | 76 (54.3) | 8 (26.6) | 0.009 |
| Diabetes mellitus (n) | 55 (39.3) | 2 (6,6) | 0.001 |
| BMI (kg/m^2^) | 47,98 (7,23) | 25,02 (3,26) | <0.0001 |
| Fasting blood sugar (mg/dL) | 112,44 (43,02) | 93,52 (13,36) | <0.0001 |
| AST (IU/L) | 22,88 (12,04) | 26,96 (22,69) | 0.199 |
| ALT (IU/L) | 29,94 (19,98) | 59,8 (135,53) | 0.283 |
| Alkaline phosphatase | 72,58 (19,42) | 87,33 (35,65) | 0.060 |
| Bilirubin (mg/dL) | 0,46 (0,26) | 0,6 (0,29) | 0.018 |
| Albumin (mg/dL) | 4,3 (0,3) | 4,42 (0,51) | 0.284 |
| Total cholesterol (mg/dL) | 187,72 (35,87) | 195 (46,9) | 0.498 |
| Triglycerides (mg/dL) | 138,18 (68,07) | 122,09 (50,17) | 0.297 |
| LDL-cholesterol (mg/dL) | 110,22 (33,67) | 122,35 (39,48) | 0.156 |
| HDL-cholesterol (mg/dL) | 48,52 (14,86) | 51,56 (18,4) | 0.493 |

Variables are presented as mean (standard deviation) or absolute frequency (percentage) and are compared by means of Mann-Whitney U test or χ^2^ test. BMI: body mass index. AST: aspartate aminotransferase. ALT: alanine aminotransferase.
