# Supplementary material for: p38α blocks brown adipose tissue thermogenesis through p38δ inhibition
Source: PLoS Biol. 2018 Jul 6;16(7):e2004455. doi: 10.1371/journal.pbio.2004455 (PMC6051667; doi:10.1371/journal.pbio.2004455)
Supplement: S3 Table — (DOCX) [file pbio.2004455.s014.docx]

**Table S3. Primers used for gene amplification**

| **Gene** | **Forward** | **Reverse** |
| --- | --- | --- |
| *Gapdh* | TGAAGCAGGCATCTGAGGG | CGAAGGTGGAAGAGTGGGA |
| *Ucp1* | GTGAACCCGACAACTTCCGAA | TGAAACTCCGGCTGAGAAGAT |
| *Ppargc1a* | TATGGAGTGACATAGAGTGTGCT | CCACTTCAATCCACCCAGA |
| *Ppargc1b* | GCTCTGACGCTCTGAAGGAC | AAGGGCTTGGGCAATCCTC |
| *Cidea* | TGACATTCATGGGATTGCAGAC | GGCCAGTTGTGATGACTAAGAC |
| *Cox7a1* | GCTCTGGTCCGGTCTTTTAGC | GTACTGGGAGGTCATTGTCGG |
| *Cox7a2* | GCTGGCCCTTCGTCAGATT | GGCATCCCATTATCCTCCTGAA |
| *Cox8b* | TGTGGGGATCTCAGCCATAGT | AGTGGGCTAAGACCCATCCTG |
| *Prdm16* | CCACCAGCGAGGACTTCAC | GGAGGACTCTCGTAGCTCGAA |
| *CoII* | CTACAAGACGCCACAT | GAGAGGGGAGAGCAAT |
| *Sdh1* | TACTACAGCCCCAAGTCT | TGGACCCATCTTCTATGC |
| *Leptin* | GAGACCCCTGTGTCGGTTC | CTGCGTGTGTGAAATGTCATT |
| *Plin1* | ACAGCAGAATATGCCGCCAA | GGCTGACTCCTTGTCTGGTG |
| *Pepck* | CCATCACCTCCTGGAAGAACA | ACCCTCAATGGGTACTCCTTC |
| *G6pc* | CGACTCGCTATCTCCAAGTGA | GTTGAACCAGTCTCCGACCA |
| *Gys2* | CCAGCTTGACAAGTTCGACA | CCTCTTCAGCATGTGCTCTG |
| *Cpt1a* | CTCCGCCTGAGCCATGAAG | CACCAGTGATGATGCCATTCT |
| *Cpt1b* | GCACACCAGGCAGTAGCTTT | CAGGAGTTGATTCCAGACAGGTA |
| *Cpt2* | CAGCACAGCATCGTACCCA | TCCCAATGCCGTTCTCAAAAT |
| *Acox1* | CCGCCACCTTCAATCCAGAG | CAAGTTCTCGATTTCTCGACG |
| *Ppardelta* | CTCGTACTTGAGCTTCATGCG | GAGCACACCCTTCCTTCCAG |
| *Ppargamma* | TCGCTGATGCACTGCCTATG | GAGAGGTCCACAGAGCTGATT |
| *Fasn* | GCGGGTTCGTGAAACTGATAA | GCAAAATGGGCCTCCTTGATA |
| *Acaca* | GATGAACCATCTCCGTTGGC | GACCCAATTATGAATCGGGAGTG |
| *Elovl6* | GAGCAGAGGCGCAGAGAAC | ATGCCGACCACCAAAGATAA |
| *Scd1* | TTCTTGCGATACACTCTGGTGC | CGGGATTGAATGTTCTTGTCGT |
| *Dgat1* | TCCGTCCAGGGTGGTAGTG | TGAACAAAGAATCTTGCAGACGA |
| *Dgat2* | GCGCTACTTCCGAGACTAC | GGGCCTTATGCCAGGAAACT |
| *Adipoq* | TGTTCCTCTTATCCTGCCCA | CCAACCTGCACAAGTTCCCTT |
| *Mapk14* | TGACCCTTATGACCAGTCCTT | GTCAGGCTCTTCCACTCATCT |
| *Mapk11* | GCGGGATTCTACCGGCAAG | GAGCAGACTGAGCCGTAGG |
| *Mapk12* | AAGGGCTTTTACCGCCAGG | GGCGCAACTCTCTGTAGGC |
| *Mapk13* | ATGAGCCTCACTCGGAAAAGG | GCATGTGCTTCAAGAGCAGAA |
| *Human Gapdh* | CCATGAGAAGTATGACAACAG | GGGTGCTAAGCAGTTGGTG |
| *Human Mapk14* | TGACACAAAAACGGGGTTACG | GGTCTGGAGAGCTTCTTCACT |
| *Human Ucp1* | AGGATCGGCCTCTACGACAC | GCCCAATGAATACTGCCACTC |
