# Supplementary material for: p38α blocks brown adipose tissue thermogenesis through p38δ inhibition
Source: PLoS Biol. 2018 Jul 6;16(7):e2004455. doi: 10.1371/journal.pbio.2004455 (PMC6051667; doi:10.1371/journal.pbio.2004455)
Supplement: S1 Text — (DOCX) [file pbio.2004455.s016.docx]

**Figure S1. Deletion of p38α in white and brown adipose tissue from p38α^Fab-KO^** **mice.**

**(a)** Western blot analysis of p38α expression in brown adipose tissue (BAT), epididymal white adipose tissue (eWAT), spleen, bone marrow (BM) and liver isolated from p38α^Fab-KO^ and control (Fab-Cre) mice. **(b)** Western blot analysis of p38α expression in bone marrow (BM), macrophages (Mɸ), neutrophils (Neutros), and monocytes (Mono). Macrophages and neutrophils were sorted from spleen, and monocytes from BM by fluorescence assisted cell sorting (FACS).
