# Supplementary material for: p38α blocks brown adipose tissue thermogenesis through p38δ inhibition
Source: PLoS Biol. 2018 Jul 6;16(7):e2004455. doi: 10.1371/journal.pbio.2004455 (PMC6051667; doi:10.1371/journal.pbio.2004455)
Supplement: S2 Text — (DOCX) [file pbio.2004455.s017.docx]

**Figure S2. Lower fat mass and improved glucose tolerance in ND-fed p38α^Fab-KO^** **mice.**

**(a)** Body weight time course in Fab-Cre and p38α^Fab-KO^ male mice (8–10-wk-old) fed a chow diet (ND) over 8 weeks. Data are presented as the increase above initial weight (mean±SEM, Fab-Cre n=9 mice; p38α^Fab-KO^ n=8 mice). **(b)** NMR analysis of fat mass in p38α^Fab-KO^ and Fab-Cre mice after 8 weeks of ND (mean±SEM, Fab-Cre n=9 mice; p38α^Fab-KO^ n=7 mice). **(c)** Weight of epidydimal white fat (eWAT), perirenal fat (pWAT), subcutaneous fat (sWAT), inguinal fat (iWAT), brown adipose tissue (BAT) and liver relativized to tibia length. (mean±SEM, Fab-Cre n=8 mice; p38α^Fab-KO^ n=7 mice). **(d)** Fasting and fed blood glucose in Fab-Cre and p38α^Fab-KO^ mice fed ND (8 wk) (mean±SEM, Fab-Cre n=9 mice; p38α^Fab-KO^ n=8 mice). **(e)** Glucose tolerance test (GTT), and insulin tolerance test (ITT) in Fab-Cre and p38α^Fab-KO^ mice fed 8 weeks the HFD. Mice were fasted overnight (for GTT) or 1 h (for ITT) and blood glucose concentration was measured in mice given intraperitoneal injections of glucose (1 g/kg of total body weight) or insulin (0.75 U/kg of total body weight). (mean±SEM, Fab-Cre n=9 mice; p38α^Fab-KO^ n=8 mice). **(f)** Immunohistochemistry of eWAT sections using anti-Glut4 (green), and anti-Caveolin-1 (red) antibodies and the nuclear dye DAPI (blue). Location of Glut4 was analysed in mice treated without or with insulin (1.5 I.U./kg) for 15 min after overnight fasting. Scale bar: 20 µm. **(g)** Comparison of energy balance between ND-fed Fab-Cre and p38α^Fab-KO^ mice. ND-fed mice were examined in a metabolic cage over a 3-day period to measure energy expenditure (EE). EE levels corrected by lean mass (left panel), expressed as ANCOVA analysis (right panel) and hour by hour over 48h-period (lower panel) are shown (mean±SEM, Fab-Cre n=9 mice; p38α^Fab-KO^ n=7 mice). **(h)** Body temperature of ND-fed Fab-Cre and p38α^Fab-KO^ mice (mean±SEM, Fab-Cre n=7 mice; p38α^Fab-KO^ n=5 mice). *p < 0.05; **p < 0.01; *** p < 0.001 Fab-Cre vs p38α^Fab-KO^ (2-way ANOVA coupled to Bonferroni’s post-tests or *t*-test or Welch’s test when variances were different). See also S1 Data.
