# Supplementary material for: p38α blocks brown adipose tissue thermogenesis through p38δ inhibition
Source: PLoS Biol. 2018 Jul 6;16(7):e2004455. doi: 10.1371/journal.pbio.2004455 (PMC6051667; doi:10.1371/journal.pbio.2004455)
Supplement: S3 Text — (DOCX) [file pbio.2004455.s018.docx]

**Figure S3. ND-fed p38α^Fab-KO^** **mice present increased expression of metabolic genes.**

**(a)** qRT-PCR analysis of mRNA expression of browning, adipogenic, glycolytic, β-oxidation, and lipogenic genes from BAT of ND-fed Fab-Cre and p38α^Fab-KO^ mice. mRNA expression was normalized to the amount of *Gapdh* mRNA. **(b)** Immunoblot analysis of PGC1α protein levels in BAT of ND-fed Fab-Cre and p38α^Fab-KO^ mice **(c)** qRT-PCR analysis of mRNA expression of browning, adipogenic, glycolytic, β-oxidation, and lipogenic genes from eWAT of ND-fed Fab-Cre and p38α^Fab-KO^ mice. mRNA expression was normalized to the amount of *Gapdh* mRNA (mean±SEM, Fab-Cre n=7 mice; p38α^Fab-KO^ n=7 mice). *p < 0.05; **p < 0.01. Fab-Cre vs p38α^Fab-KO^ (*t*-test or Welch’s test when variances were different). See also S1 Data.
