# Supplementary material for: p38α blocks brown adipose tissue thermogenesis through p38δ inhibition
Source: PLoS Biol. 2018 Jul 6;16(7):e2004455. doi: 10.1371/journal.pbio.2004455 (PMC6051667; doi:10.1371/journal.pbio.2004455)
Supplement: S4 Text — (DOCX) [file pbio.2004455.s019.docx]

**Figure S4. HFD-fed p38α^Fab-KO^** **mice are protected against diet-induced diabetes.**

Fab-Cre and p38α^Fab-KO^ mice were fed a HFD for 8 weeks. **(a)** Weight of epididymal white fat (eWAT), subcutaneous WAT (sWAT), inguinal fat (iWAT), perirenal WAT (pWAT), brown adipose tissue (BAT) and liver relativized to tibia length. (mean±SEM, Fab-Cre n=10 mice; p38α^Fab-KO^ n=8 mice). **(b)** Glucose tolerance test (GTT) in Fab-Cre and p38α^Fab-KO^ mice fed 8 weeks the HFD. Mice were fasted overnight and blood glucose concentration was measured in mice given intraperitoneal injections of glucose (1 g/kg of lean mass). (mean±SEM, Fab-Cre n=5 mice; p38α^Fab-KO^ n=6 mice). **(c)** Western blot analysis of Akt activation in the liver from Fab-Cre mice fed with ND or HFD. Mice were treated without or with insulin (1.5 I.U./kg) for 15 min after overnight fasting. Each line represents a different mouse. **(d)** Western blot analysis of Akt activation in the liver, skeletal muscle, eWAT and BAT from mice fed with HFD. Mice were treated without or with insulin (1.5 I.U./kg) for 15 min after overnight fasting. Each line represents a different mouse. **(e)** Triglyceride content in blood samples from Fab-Cre and p38α^Fab-KO^ mice (mean±SEM, Fab-Cre n=12 mice; p38α^Fab-KO^ n=8 mice). *p < 0.05, ***p < 0.001 Fab-Cre vs p38α^Fab-KO^ (2-way ANOVA coupled to Bonferroni’s post-tests or *t*-test or Welch’s test when variances were different). See also S1 Data.
