# Supplementary material for: p38α blocks brown adipose tissue thermogenesis through p38δ inhibition
Source: PLoS Biol. 2018 Jul 6;16(7):e2004455. doi: 10.1371/journal.pbio.2004455 (PMC6051667; doi:10.1371/journal.pbio.2004455)
Supplement: S5 Text — (DOCX) [file pbio.2004455.s020.docx]

**Figure S5. Fat depots from** **HFD-fed p38α^Fab-KO^** **mice present smaller adipocytes.**

Fab-Cre and p38α^Fab-KO^ mice were fed a HFD for 8 weeks. **(a)** Immunohistochemistry of eWAT sections using anti-Ki67 (red), and anti-perilipin (green) antibodies and the nuclear dye DAPI (blue) (upper panel). Scale Bar: 20 µm. A positive cell is shown in a bigger magnification for each genotype. Quantification of proliferation and adipocyte size are shown (lower panel) (mean±SEM, Fab-Cre n=5 mice; p38α^Fab-KO^ n=5 mice and 5 pictures of each mouse). **(b)** Staining of UCP1 after 8 weeks of HFD in eWAT. Representative pictures are shown from Fab-Cre n=6 mice; p38α^Fab-KO^ n=6 mice with 3 pictures of each mouse. Scale Bar: 50 µm. **(c)** Immunohistochemistry of BAT sections using anti-Ki67 (red), and anti-perilipin (green) antibodies and the nuclear dye DAPI (blue) (upper panel). Scale Bar: 20 µm. A positive cell is shown in a bigger magnification for each genotype. Quantification of proliferation and adipocyte size are shown (lower panel). (mean±SEM, Fab-Cre n=5 mice; p38α^Fab-KO^ n=5 mice and 5 pictures of each mouse). *p < 0.05, ***p < 0.001 Fab-Cre vs p38α^Fab-KO^ (*t*-test or Welch’s test when variances were different). See also S1 Data.
