# Supplementary material for: p38α blocks brown adipose tissue thermogenesis through p38δ inhibition
Source: PLoS Biol. 2018 Jul 6;16(7):e2004455. doi: 10.1371/journal.pbio.2004455 (PMC6051667; doi:10.1371/journal.pbio.2004455)
Supplement: S6 Text — (DOCX) [file pbio.2004455.s021.docx]

**Figure S6. Specificity of UCP1 antibody.**

Western blot analysis of UCP1 in eWAT from Fab-Cre and p38α^Fab-KO^ mice fed with a high fat diet (HFD). BAT from control mice (dil 1/10) was used as positive control. Non-diluted BAT and eWAT from UCP1^-/-^ mice were used as negative controls. Each line represents a different mouse. Two different exposures are showed.
