# Supplementary material for: p38α blocks brown adipose tissue thermogenesis through p38δ inhibition
Source: PLoS Biol. 2018 Jul 6;16(7):e2004455. doi: 10.1371/journal.pbio.2004455 (PMC6051667; doi:10.1371/journal.pbio.2004455)
Supplement: S7 Text — (DOCX) [file pbio.2004455.s022.docx]

**Figure S7. HFD-fed p38α^Fab-KO^** **mice have higher iWAT and lower eWAT browning.**

Fab-Cre and p38α^Fab-KO^ mice were fed with HFD for 8 weeks. Immunoblot analysis of UCP1 protein levels and Creb, ATF2, p38, AMPK and ACC phosphorylation in lysates from inguinal WAT (iWAT) **(a)** or epididymal WAT (eWAT) **(b).** Quantifications are shown in lower panels (mean±SEM, Fab-Cre n=4-10 mice; p38α^Fab-KO^ n=4-10 mice). *p < 0.05 Fab-Cre vs p38α^Fab-KO^ (*t*-test or Welch’s test when variances were different). See also S1 Data
