# Supplementary material for: p38α blocks brown adipose tissue thermogenesis through p38δ inhibition
Source: PLoS Biol. 2018 Jul 6;16(7):e2004455. doi: 10.1371/journal.pbio.2004455 (PMC6051667; doi:10.1371/journal.pbio.2004455)
Supplement: S8 Text — (DOCX) [file pbio.2004455.s023.docx]

**Figure S8. p38α controls brown adipocyte differentiation *in vitro.***

**(a-e)** Primary adipocytes isolated from interscapular BAT of Fab-Cre and p38α^Fab-KO^ were differentiated *in vitro*. **(a)** Immunoblot analysis of PGC1α and UCP1 protein levels (left panel) and qRT-PCR analysis of browning genes mRNA expression (right panel). mRNA expression was normalized to the amount of *Gapdh* mRNA. (mean±SEM, Fab-Cre n=6 mice; p38α^Fab-KO^ n=6 wells from 2 independent experiments). qRT-PCR analysis of mRNA expression of adipogenic **(b)**, glycolytic **(b)**, β-oxidation **(c)**, and lipogenic **(c)** genes in *in vitro* differentiated primary brown adipocytes (BA). mRNA expression was normalized to the amount of *Gapdh* mRNA. (mean±SEM, a representative experiment is shown. Fab-Cre n=6 wells; p38α^Fab-KO^ n=6 wells). **(d)** Oil Red O staining of primary BA after 10 days of differentiation *in vitro*. **(e)** Confocal imaging of Fab-Cre and p38α^Fab-KO^ primary BA stained with Mitotracker Deep Red (red) and Bodipy (green). Scale bar: 10 µm (left panel). Quantification of cellular triglyceride content in *in vitro* differentiated primary BA (right panel) (mean±SEM, a representative experiment is shown. Fab-Cre n=4 wells; p38α^Fab-KO^ n=5 wells). Statistically significant differences between Fab-Cre and p38α^Fab-KO^ BA are indicated: *p < 0.05; **p < 0.01; *** p < 0.001 (*t*-test or Welch’s test when variances were different). **(f, g)** Primary adipocytes isolated from subcutaneous white fat of Fab-Cre and p38α^Fab-KO^ were differentiated *in vitro*. **(f)** qRT-PCR analysis of mRNA expression of adipogenic genes in *in vitro* differentiated primary white adipocytes (WA). mRNA expression was normalized to the amount of *Gapdh* mRNA. (mean±SEM, a representative experiment is shown. Fab-Cre n=9 wells; p38α^Fab-KO^ n=8 wells from 3 independent experiments) *p < 0.05 (Welch’s test). **(g)** Oil Red O staining of primary WA after 9 days of differentiation *in vitro*. See also S1 Data.
