# Supplementary material for: p38α blocks brown adipose tissue thermogenesis through p38δ inhibition
Source: PLoS Biol. 2018 Jul 6;16(7):e2004455. doi: 10.1371/journal.pbio.2004455 (PMC6051667; doi:10.1371/journal.pbio.2004455)
Supplement: S9 Text — (DOCX) [file pbio.2004455.s024.docx]

**Figure S9. Activation of p38 isoforms in Fab-Cre and p38α^Fab-KO^ mice after high-fat diet.**

**(a)** Phosphorylation of p38 isoforms in adipocytes detected with cell signal antibody #9211. Western blot analysis of the different p38 isoforms activation in adipocytes from WT and p38γ/δ^-/-^ cells. **(b)** Immnoblot analysis of p38 phosphorilation in brown fat (BAT), epididymal white fat (eWAT), inguinal WAT (iWAT), subcutaneous WAT (sWAT), and perirenal WAT (pWAT) lysates from ND-fed Fab-Cre and p38α^Fab-KO^ mice. **(c)** Effect of SB203580 on phosphorylation of p38 isoforms. Western blot analysis of phospho p38 in brown pre-adipocytes from Fab-Cre mice treated with DMSO, sorbitol (0.5M, 15 min) or sorbitol with SB 203580 (10 µM, 1h pre-treatment) or from p38α^Fab-KO^ mice with DMSO. **(d)** qRT-PCR analysis of different isoforms of p38 mRNA expression (p38α (*Mapk14*), p38β (*Mapk11*), p38γ (*Mapk12*), p38δ (*Mapk13*)) in BAT and eWAT from control mice (Fab-Cre) after a ND or a HFD for 8 weeks. mRNA expression was normalized to the amount of *Gapdh* mRNA. (mean±SEM, ND n=6-9 mice; HFD n=14 mice). **(e)** Comparison of p38 isoforms mRNA expression by qRT-PCR analysis in BAT from ND-fed Fab-Cre and p38α^Fab-KO^ mice. mRNA expression was normalized to the amount of *Gapdh* mRNA (mean±SEM, Fab-Cre n=6 mice; p38α^Fab-KO^ n=7 mice). **(f)** Comparison of p38 isoforms mRNA expression by qRT-PCR analysis in eWAT from ND and HFD-fed Fab-Cre and p38α^Fab-KO^ mice. mRNA expression was normalized to the amount of *Gapdh* mRNA (mean±SEM, Fab-Cre n=7-14 mice; p38α^Fab-KO^ n=7-9 mice).*p < 0.05; **p < 0.01; *** p < 0.001; Fab-Cre vs p38δ^Fab-KO^ (*t*-test or Welch’s test when variances were different). See also S1 Data.
