# Supplementary material for: p38α blocks brown adipose tissue thermogenesis through p38δ inhibition
Source: PLoS Biol. 2018 Jul 6;16(7):e2004455. doi: 10.1371/journal.pbio.2004455 (PMC6051667; doi:10.1371/journal.pbio.2004455)
Supplement: S10 Text — (DOCX) [file pbio.2004455.s025.docx]

**Figure S10. Brown fat from p38δ^Fab-KO^ mice presents a decrease in BAT activity.**

Fab-Cre and p38δ^Fab-KO^ mice were fed with a ND for 8 weeks. **(a)** Weight of epididymal white fat (eWAT), subcutaneous WAT (sWAT), inguinal WAT (iWAT), perirenal WAT (pWAT), brown fat (BAT), and liver with respect to tibia length in ND-fed Fab-Cre and p38δ^Fab-KO^ mice (mean±SEM, Fab-Cre n=6 mice; p38δ^Fab-KO^ n=6 mice). **(b)** qRT-PCR analysis of mRNA expression of browning genes in BAT isolated from ND-fed Fab-Cre and p38δ^Fab-KO^ mice. mRNA expression was normalized to the amount of *Gapdh* mRNA. **(c)** Western blot analysis of PKA activation in BAT from Fab-Cre and p38δ^Fab-KO^. Each line represents a different mouse. (n=6) (mean±SEM, Fab-Cre n=6 mice; p38δ^Fab-KO^ n=6 mice). *p < 0.05; **p < 0.01; Fab-Cre vs p38δ^Fab-KO^ (*t*-test or Welch’s test when variances were different). See also S1 Data.
