# Supplementary material for: p38α blocks brown adipose tissue thermogenesis through p38δ inhibition
Source: PLoS Biol. 2018 Jul 6;16(7):e2004455. doi: 10.1371/journal.pbio.2004455 (PMC6051667; doi:10.1371/journal.pbio.2004455)
Supplement: S11 Text — (DOCX) [file pbio.2004455.s026.docx]

**Figure S11. p38δ^Fab-KO^ mice have higher body weight and lower temperature when fed a high-fat diet.**

Fab-Cre and p38δ^Fab-KO^ mice were fed with a HFD for 8 weeks. **(a)** Body weight at the end of the treatment. (mean±SEM, Fab-Cre n=8 mice; p38δ^Fab-KO^ n=7 mice). **(b)** NMR analysis of body mass and fat mass in p38δ^Fab-KO^ and Fab-Cre mice after 8 weeks of HFD (mean±SEM, Fab-Cre n=8 mice; p38δ^Fab-KO^ n=7 mice). **(c)** Weight of epididymal white fat (eWAT), subcutaneous WAT (sWAT), inguinal WAT (iWAT), perirenal WAT (pWAT), brown fat (BAT), and liver with respect to tibia length (mean±SEM, Fab-Cre n=8 mice; p38δ^Fab-KO^ n=7 mice). **(d)** Skin temperature surrounding interscapular BAT in HFD-fed Fab-Cre and p38δ^Fab-KO^. Right panels show representative infrared thermal images (mean±SEM, Fab-Cre n=8 mice; p38δ^Fab-KO^ n=7 mice). **(e)** qRT-PCR analysis of mRNA expression of browning genes in BAT isolated from HFD-fed Fab-Cre and p38δ^Fab-KO^ mice. mRNA expression was normalized to the amount of *Gapdh* mRNA. (mean±SEM, Fab-Cre n=5 mice; p38δ^Fab-KO^ n=6 mice). **(f)** Immunoblot of UCP1 protein levels in p38δ^Fab-KO^ and Fab-Cre mice after 8 weeks of HFD. Quantification is shown on the right panel (mean±SEM, Fab-Cre n=5 mice; p38δ^Fab-KO^ n=6 mice). *p < 0.05; **p < 0.01; ***p < 0.001; Fab-Cre vs p38δ^Fab-KO^ (*t*-test or Welch’s test when variances were different). See also S1 Data.
